# Receptiveness of physicians towards artificial intelligence-driven drug prescription: a nationwide survey

**DOI:** 10.1038/s44401-026-00101-3

**Published:** 2026-06-08

**Authors:** Wei Zhang, Saibing Qi, Yu Hu, Xiaolin Zhai, Wangsong Zhai, Sichang Liu, Zhenwei Li, Wei Su, Simiao Liu, Wu Liu, Jie Liu, Jiejing Yin, Mengyi Xie, Ang Zheng, Lanying Zhang, Ai He, Ruifen Zhang, Weilin Liu, Kan Ding, Lianzhong Wu, Yongsheng Meng, Qi Li, Baima Yangjin, Ting Li, Chao Liu, Da Gao, Heng He, Fei Long, Xiaoyan Ge, Xiao-Xiao Sun, Haizhao Ma, Tiansheng Su, Shuhao Du, Mengyun Chen, Yahui Feng, Zhen Song, Jinyu Wang, Robert Peter Gale, Xiaowen Gong, Qiujin Shen, Junren Chen

**Affiliations:** 1 State Key Laboratory of Experimental Hematology, National Clinical Research Center for Blood Diseases, Haihe Laboratory of Cell Ecosystem, Institute of Hematology & Blood Diseases Hospital, Chinese Academy of Medical Sciences & Peking Union Medical College, Tianjin, China; 2 Tianjin Institutes of Health Science, Tianjin, China; 3 Tianjin Jinghai District Hospital, Tianjin, China; 4 Qinghai Red Cross Hospital, Xining, China; 5 Hospital of Integrated Chinese and Western Medicine, Tianjin, China; 6 Jinzhai County Hospital of Traditional Chinese Medicine, Liuan, China; 7 Sinopharm Tongmei Central Hospital, Datong, China; 8 The Second Hospital of Traditional Chinese Medicine of Kaifeng, Kaifeng, China; 9 The Second Affiliated Hospital of Fujian Medical University, Quanzhou, China; 10 The Second Affiliated Hospital of Xuzhou Medical University, Xuzhou, China; 11 The Second Hospital of Hebei Medical University, Shijiazhuang, China; 12 The First Affiliated Hospital of Guangxi University of Chinese Medicine, Nanning, China; 13 Inner Mongolia Autonomous Region Hospital of Traditional Chinese Medicine, Hohhot, China; 14 Deyang People’s Hospital, Deyang, China; 15 The First Affiliated Hospital of Anhui University of Chinese Medicine, Hefei, China; 16 Huzhu Tu Ethnicity County People’s Hospital, Haidong City, China; 17 Central Hospital of Kaifeng, Kaifeng, China; 18 Liaoning Electric Power Center Hospital, Shenyang, China; 19 Xizang Autonomous Region People’s Hospital, Lhasa, China; 20 Huzhou Hospital of Traditional Chinese Medicine Affiliated to Zhejiang University of Traditional Chinese Medicine, Huzhou, China; 21 Anhui Campus of the Second Affiliated Hospital, Zhejiang University School of Medicine, Bengbu, China; 22 The First Affiliated Hospital of Bengbu Medical College, Bengbu, China; 23 The Affiliated Hospital of Inner Mongolia Medical University, Hohhot, China; 24 Feidong County People’s Hospital, Hefei, China; 25 Xuancheng People’s Hospital, Xuancheng, China; 26 Second Hospital of Shanxi Medical University, Taiyuan, China; 27 Tianjin First Central Hospital, Tianjin, China; 28 The Fifth People’s Hospital of Qinghai Province, Xining, China; 29 Second Affiliated People’s Hospital of Fujian University of Traditional Chinese Medicine, Fuzhou, China; 30 First Teaching Hospital of Tianjin University of Traditional Chinese Medicine, Tianjin, China; 31 Centre for Haematology, Department of Immunology and Inflammation, Imperial College of Science, Technology and Medicine, London, UK

**Keywords:** Computational biology and bioinformatics, Health care, Mathematics and computing, Medical research

## Abstract

Using artificial intelligence (AI) to prescribe drugs has advanced slowly. Whether a “doctor-in-the-loop” design would increase acceptance of drug-prescribing AI is unknown, as are settings where physicians envision AI-driven drug prescription most likely to be implemented. We surveyed a stratified sample of 2708 physicians throughout China to interrogate their opinions on drug-prescribing AI. Most respondents (78%) are receptive to using drug-prescribing AI and anticipate doing so within 5 years. Respondents suggested initial settings for AI-driven drug prescribing include situations where there are standard guidelines (74%), where the decision is whether to continue a current prescription in someone (55%), and where prescribing decisions rely on high-complexity clinical data (44%). Many (66%) indicated a preference for conditional to fully autonomous drug-prescribing AI. Clustering analysis identified 2 psychological profile-types, “optimists” and “pragmatists”, who have different standards for model efficacy, expediency, explainability, and governance/stewardship for drug-prescribing AI. A high level of using medical AI is the strongest predictor for being an optimist (OR = 2.98 [2.53, 3.51]; *P* < 0.0001). In conclusion, our data point to the wide acceptability of conditional autonomous drug-prescribing AI among Chinese physicians. Moreover, disparity in optimism about drug-prescribing AI is caused by disparity in prior exposure to medical AI.

## Introduction

Use of artificial intelligence (AI) are rapidly increasing in several spheres of healthcare, including electronic health records, diagnostic imaging, radiation therapy planning, robotic surgery, brain implants, wearable technology, chatbots, and others. However, using AI to prescribe drugs has advanced only slowly^1–4^. Physician resistance to drug-prescribing AI is perceived to be an important obstacle^2,3^. Potential variables influencing this resistance, like sex, age, highest education degree, workload, clinical specialty, clinical practicing experience, physician rank, experience with medical AI, hospital tier, and economic development level of a geographic region, are poorly understood. Whether a “doctor-in-the-loop” design—that is, making AI-driven drug prescription conditional on physicians’ discretion—would increase acceptance of drug-prescribing AI is also unknown, as are settings where physicians envision drug-prescribing AI is most likely to be implemented. How physicians’ decision-making processes or values might interact or clash with AI’s is also poorly known^5,6^.

Against the backdrop of progress China has made in medical AI in recent years, attitudes of Chinese physicians towards this issue may provide insights^7^. In a recent prospective study we conducted, an autonomous conditional drug-prescribing AI model was deployed to assist transplant experts in making complex decisions on a pre-emptive drug intervention to prevent severe acute graft-versus-host disease (GvHD) in persons receiving a human leukocyte antigen (HLA)-haplotype-matched haematopoietic cell transplant^8,9^. Most physicians and patients agreed to let AI prescribe a drug in this setting, and compliance was high. However, it is unknown whether physicians are receptive to drug-prescribing AI in more common clinical settings.

To address this knowledge gap, we designed a large-scale, quantitative survey study that used a stratified sampling strategy to interrogate Chinese physicians regarding their opinions on drug-prescribing AI^10^. We found most respondents receptive to using drug-prescribing AI and anticipating using it within 5 years. Most respondents prefer conditional to fully autonomous drug-prescribing AI. Respondents suggested initial settings for AI-driven drug prescription include situations where there is little ambiguity in a prescription and situations where prescribing decisions rely on high-complexity clinical data. Male physicians are more optimistic about drug-prescribing AI compared with female physicians, a disparity mediated by differences in medical AI use experience between the sexes.

## Results

### Stratified sampling of participants

Survey was conducted during 16 May 2025–29 September 2025 in 14 provincial-level administrative divisions (“provinces”) of China. Per capita gross domestic product (GDP) in 2024 was > 15,000 US dollars ($) in 5 of the surveyed provinces (Jiangsu, Fujian, Zhejiang, Tianjin, Inner Mongolia), $10,001 –15,000 in 5 provinces (Anhui, Liaoning, Sichuan, Tibet, Shanxi), and $7001–10,000 in 4 (Qinghai, Henan, Hebei, Guangxi)^11^.

**Fig. 1:**
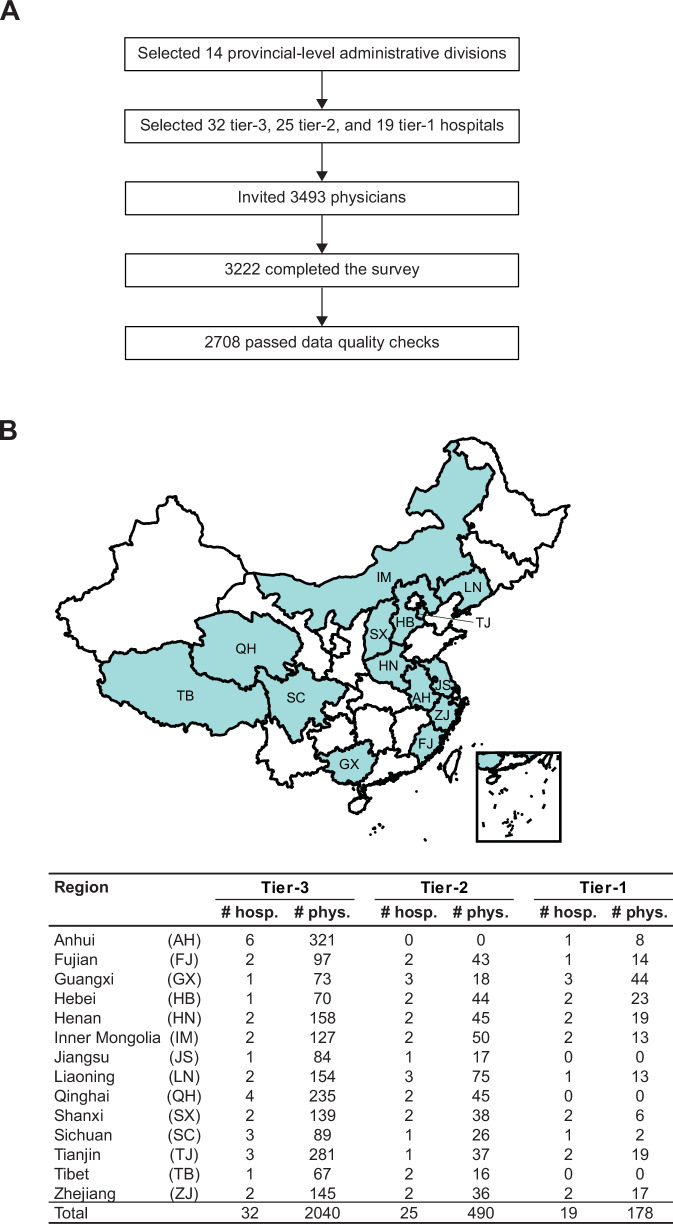
A stratified sample of 2708 physicians. **A** Work flow for stratified sampling and data quality control. **B** Distribution of the 2708 respondents whose returned questionnaires passed data quality checks. “hosp.” and “phys.” stand for hospital(s) and physician(s), respectively.

3493 physicians, including 2632 drawn from 32 tier-3 (highest-level) hospitals, 614 from 25 tier-2 hospitals, and 247 from 19 tier-1 (lowest-level) hospitals, participated in the survey (Fig. 1A; “Methods”). The surveyed hospitals are listed in Supplementary Table 1. 271 (7.8%) participants did not complete the survey.

Among the 3222 (92.2% of 3493) participants who completed the survey, median time to completion was 7 min (IQR, 4–12). To ensure data quality, we excluded data from 278 (8.6% of 3222) physicians who completed in <3 min. Subsequently, 236 (7.3% of 3222) filled-out questionnaires that failed to pass data quality checks were also excluded (“Methods”). The total “bad response rate” was 16.0% (514/3222; Fig. 1A).

The final dataset includes 2708 physicians (Fig. 1B). 1640 (60.6%) and 1068 (39.4%) are internists and surgeons, respectively. 980 (36.2%) are from the most economically developed provinces (Jiangsu, Fujian, Zhejiang, Tianjin, Inner Mongolia) in our sample, 954 (35.2%) are from moderately developed provinces (Anhui, Liaoning, Sichuan, Tibet, Shanxi), and 774 (28.6%) from the least developed provinces (Qinghai, Henan, Hebei, Guangxi). 2040 (75.3%), 490 (18.1%), and 178 (6.6%) respondents are from tier-3, -2, and -1 hospitals, respectively.

### Demographics of respondents

The survey consisted of 32 questions (Q) divided into multiple sections (Supplementary Table 2; “Methods”).

Q1–Q6 collected the participants’ demographic information. 383 (14.1%) are chief physicians (highest-rank), with a male-to-female ratio of 1.3 (220/163), a median age of 51 years (IQR, 47–55), and median clinical practicing experience of 27 years (IQR, 22–31). 762 (28.1%) are associate chief physicians, with male-to-female ratio 1.2 (411/351), median age 44 (IQR, 40–48), and median experience 18 years (IQR, 14–24). 1563 (55.5%) are junior attending physicians (lowest-rank), with male-to-female ratio 0.8 (670/893), median age 36 (IQR, 33–39), and median experience 10 years (IQR, 7–14). Physician rank significantly correlates with sex (*P* < 0.0001 [chi-squared]), age (Spearman’s rank correlation *ρ* = 0.68 [*P* < 0.0001]), and years of clinical practice (*ρ* = 0.66 [*P* < 0.0001]).

### Self-assessed levels of AI-related knowledge and use experience

Q7–Q10 asked the participants to self-assess their familiarity with AI and level of use experience. 1494 (55.2%) respondents indicated that they have some familiarity with AI-related knowledge and that they are confident to make decisions regarding AI technology based on their own judgment or after consulting with experts or perusing literature. 2311 (85.3%) respondents stated they have experience using medical AI. 1049 (38.7%) self-assessed they are good at using medical AI tools.

Male physicians are more likely to agree or strongly agree that they are good at using medical AI, with an odds ratio (OR) of 1.52 (95-percent confidence interval [CI], [1.29, 1.80]; *P* < 0.0001) compared with females after controlling for age, highest educational degree, years of clinical practice, weekly outpatient volume, weekly inpatient volume, physician rank, clinical specialty, hospital tier, and provincial per capita GDP.

### Outlook for the future of AI in healthcare

Q11–Q15 asked the participants to express their outlook on the future of medical AI. The majority agree or strongly agree that AI will eventually transform the healthcare industry (*n* = 1951 [72.0%]), augment physicians’ capabilities (1934 [71.4%]), improve healthcare quality (1960 [72.4%]), improve equity in access to healthcare (1812 [66.9%]), or facilitate physicians’ training (1847 [68.2%]). 1954 (72.2%) respondents agree or strongly agree with ≥3 of these statements, and they have significantly higher self-assessed level of experience using medical AI (*P* < 0.0001) compared with the other respondents (*n* = 754 [27.8%]).

Q16 was a forced-choice question requesting the participants to consider the trade-off between model efficacy and physicians’ self-autonomy. 1782 (65.8%) respondents indicated they value their own autonomy more than an AI model’s clinical efficacy, implying that they prefer conditional to fully autonomous AI.

### Projected use cases of AI-driven drug prescription

Q17 requested the participants to identify initial settings for drug-prescribing AI. 46 (1.7%) physicians responded “None”. The most commonly mentioned settings were: (1) when there exist standardized treatment guidelines, *n* = 1995 (73.7%); (2) when the decision is whether to extend a current therapy in someone, 1496 (55.2%); (3) when many variables need considering in the prescribing decision, 1198 (44.2%); and (4) when reliance solely on human decisions may cause critical delay in therapy, 660 (24.4%). Only 291 (10.7%) respondents indicated drug-prescribing AI is useful when there is a shortage of qualified staff. In other words, most physicians believe that, even with drug-prescribing AI, skilled physicians will remain indispensable.

### Perceived importance of technical attributes

Q18–Q23 interrogated how physicians perceive the relative importance of 5 technical attributes of drug-prescribing AI: vetted efficacy, expediency, transparency, explainability, and governance/stewardship. Expediency and vetted efficacy received the highest average rank (1.9 [highest = 1, lowest = 5] and 2.0, respectively), significantly higher than explainability (3.5 [*P* < 0.0001]), governance/stewardship (3.7 [*P* < 0.0001]), and transparency (3.8 [*P* < 0.0001]).

For a drug-prescribing AI model to be perceived as expedient, 1645 (60.7%) respondents stated the model must be embedded in the clinical workflow, 1324 (48.9%) stated the model should shorten physicians’ work hours without compromising quality of care, 1099 (40.6%) stated the model should enable physicians to care for more patients, and 1056 (39.0%) stated the model should not increase physicians’ workload. Few respondents anticipate that drug-prescribing Al’s expediency lies in improving patients’ trust in physicians (*n* = 29 [1.1%]) or facilitating the hiring and training of junior physicians (102 [3.8%]).

To properly vet the efficacy of drug-prescribing AI, 1306 (48.2%) stated it is important that the model is developed using data from subjects similar to patients in their own practice, and 1068 (39.4%) stated the model needs to be validated in a domestic, Chinese cohort. In terms of model explainability, 1275 (47.1%) stated they need to understand how the model converts input variables to drug-prescribing recommendations, and 1273 (47.0%) respondents emphasize the model must explain its reasoning when its recommendation is discordant with physicians’ opinions. In terms of transparency, the majority of respondents (*n* = 2184 [80.6%]) indicated they need to know at least portions of technical details in model development before they can decide whether to adopt a drug-prescribing AI model in their clinical practice.

As for model governance and stewardship, 1468 (54.2%) indicated the importance of a trustworthy mechanism for monitoring quality, bias, and safety of AI prescriptions, 1244 (45.9%) emphasize mechanisms for model maintenance and/or upgrades, and 1167 (43.1%) desire easy accessibility to technical support staffs.

### Perceived importance of institutional attributes

Q24–Q27 queried physicians’ perceived importance of hospital attributes in successful adoption of drug-prescribing AI. Most respondents indicated it is crucial that hospitals embrace new technologies (*n* = 1712 [63.2%]), value innovation (1711 [63.2%]), and invest resources in AI initiatives (1370 [50.6%]). In addition, 1358 (50.1%) stated that hospitals need to value employee satisfaction and workplace harmony, and 868 (32.1%) stated that hospitals need to promote learning and talent development.

For successful transition from the conventional drug prescribing workflow to AI-driven drug prescription, 1576 (58.2%) respondents highlighted the importance of having local “champions” for AI initiatives at hospitals, 1498 (55.3%) mentioned the importance of hospital leadership support, 1482 (54.7%) indicated that the hospital administration needs to provide an internal guidance on the use of drug-prescribing AI within the institution, and 887 (32.8%) stated that planning for the launch of drug-prescribing AI must be led by a multi-disciplinary task force.

The participants were also asked to give their opinions on what expertise(s) hospitals should focus on improving after adopting drug-prescribing AI. 1719 (63.5%) stated hospitals should improve ability to provide multi-disciplinary care to patients. 1278 (47.2%) and 1246 (46.0%) highlighted the importance of improving cost-effectiveness and throughput, respectively. 1093 (40.4%) stated hospitals should improve ability to manage difficult-to-treat diseases.

### Perceived importance of governmental attributes

Q28–Q30 queried the participants’ opinions on the government’s role in successful adoption of drug-prescribing AI. Almost half (1317 [48.6%]) of the respondents prioritize a nation-wide policy mandate to promote AI in healthcare and a nation-wide initiative to build out technological infrastructures for medical AI over a nation-wide initiative to educate and train AI-related talents. 2037 (75.2%), 1861 (68.7%), and 1840 (67.9%) mentioned that professional medical societies, regulatory authorities, and separate individual hospitals, respectively, should be partially or wholly responsible for setting the standards for AI-driven drug prescription. 1472 (54.4%), 1221 (45.1%), and 1135 (41.9%) expressed that hospitals, AI developers, and physicians, respectively, should be remunerated for AI-driven drug prescription.

### Self-assessed readiness for adopting drug-prescribing AI

Q31 and Q32 asked the participants to assess their hospitals’ and their own readiness for adopting drug-prescribing AI. 2224 (82.1%) forecasted their hospitals would be ready to adopt drug-prescribing AI within 5 years. 2117 (78.2%) forecasted they themselves would be ready to adopt within 5 years.

### Clustering analysis of psychological profile-types

**Fig. 2:**
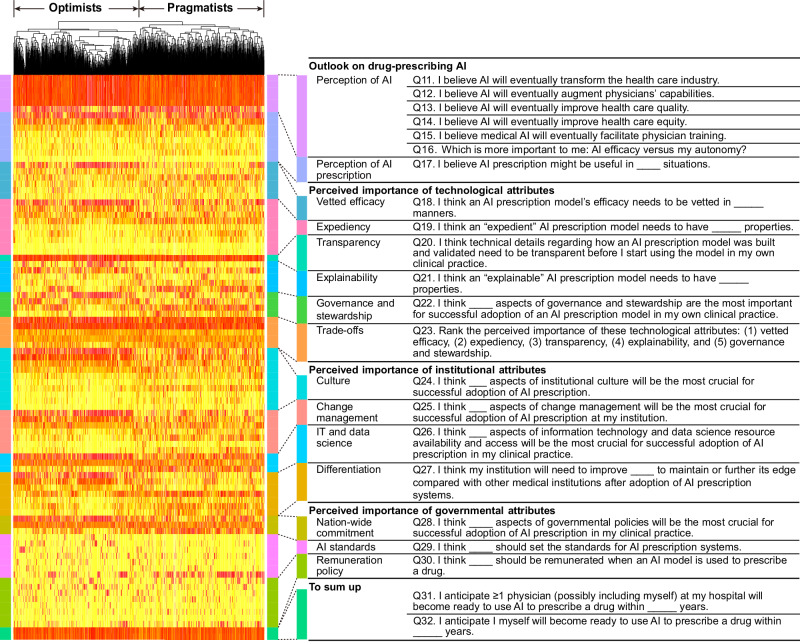
Identification of Chinese physicians’ psychological profile-types with respect to drug-prescribing artificial intelligence (AI). The heat map on the left-hand side, wherein each row represents a separate survey item and each column a separate respondent, is the result of hierarchical clustering on the 2708 respondents based on their psychological characteristics (i.e., responses to Q11 – Q32 [yellow, lower values; red, higher values]; Methods). Survey questions Q11 – Q32 are listed on the right-hand side.

Clustering analysis of the respondents’ psychological characteristics (i.e., responses to Q11 – Q32) indicated that the surveyed physicians can be classified into 2 distinct psychological profile-types (Fig. 2). We termed the 2 psychological profile-types “optimists” (*n* = 1358 [50.1%]) and “pragmatists” (1350 [49.9%]). To assess the robustness of the clustering result, we performed 1000 independent bootstrapping experiments. Optimists’ average proportion in the bootstrapping experiments was 64.5% (standard deviation [SD] = 16.2%), and pragmatists’ average proportion was 35.5% (SD = 16.2%).

How physicians of different psychological profile-types responded to survey questions is summarized in Supplementary Table 2.

There is a positive but insignificant correlation between provincial per capita GDP and the proportion of optimists among the physicians in a province: Spearman’s rank correlation *ρ* = 0.16 (*P* = 0.59). Proportions of optimists are comparable among physicians working at tier-3, -2, and -1 hospitals (51.1% [1042/2040], 46.3% [227/490], and 50.0% [89/178]; *P* = 0.17; chi-squared test). Proportions of optimists in surgeons and internists are comparable (51.6% [551/1068] vs. 49.2% [807/1640]; *P* = 0.24).

Most physicians, regardless of psychological profile-type, are receptive to using autonomous drug-prescribing AI in the near future. However, a higher proportion of optimists, compared with pragmatists, forecasted they will be ready to adopt drug-prescribing AI within 5 years (81.5% [1107/1358] vs. 74.8% [1010/1350]; *P* < 0.0001).

A higher proportion of optimists agree or strongly agree that AI will eventually transform the healthcare industry (79.7% [1082/1358] vs. 64.4% [869/1350]; *P* < 0.0001 [chi-squared]), augment physicians’ capabilities (79.8% [1084/1358] vs. 63.0% [850/1350]; *P* < 0.0001), improve healthcare quality (80.9% [1098/1358] vs. 63.9% [862/1350]; *P* < 0.0001), improve equity in access to healthcare (77.0% [1046/1358] vs. 56.7% [766/1350]; *P* < 0.0001), or facilitate physicians’ training (78.8% [1070/1358] vs. 57.6% [777/1350]; *P* < 0.0001) compared with the pragmatists. Single-variable logistic regression indicated that optimists are significantly more likely to value model efficacy above physicians’ self-autonomy (OR = 1.65; 95-percent Cl, [1.40, 1.94]; *P* < 0.0001) compared with the pragmatists.

Optimists and pragmatists have different priorities and expectations for drug-prescribing AI models. A significantly higher proportion of optimists, compared with pragmatists, stated that drug-prescribing AI would be most useful when there are standard treatment plans according to clinical guidelines (80.8% [1097/1358] vs. 66.5% [898/1350]; *P* < 0.0001). Optimists are also more tolerant of “black-box” AI models and therefore less likely than pragmatists to strongly insist AI explain the rationale underlying the prescription it recommends (12.6% [171/1358] vs. 21.5% [290/1350]; *P* < 0.0001). When considering the expediency of a drug-prescribing AI model, the majority (74.6% [1013/1358]) of optimists insist the model be embedded in the clinical workflow, whilst most (58.9% [795/1350]) pragmatists stated the model should shorten physicians’ work hours. With respect to vetting a drug-prescribing AI model’s efficacy, optimists focus more on the characteristics of patient cohorts used to develop and test the model. Optimists, compared with pragmatists, rely less on endorsements from high-impact-factor academic journals (20.0% [272/1358] vs. 33.3% [450/1350]; *P* < 0.0001), international medical professional societies (15.1% [205/1358] vs. 24.8% [335/1350]; *P* < 0.0001), or domestic medical societies (11.2% [152/1358] vs. 20.1% [271/1350]; *P* < 0.0001). In terms of model explainability, most (57.1% [775/1358]) optimists indicated that it is important they understand how input variables are converted to model outputs, whilst the majority (64.9% [876/1350]) of pragmatists insist the model explain its reasoning when giving a recommendation discordant with their opinions. Regarding model governance and stewardship, optimists emphasize a trustworthy mechanism for model maintenance and upgrades (60.2% [817/1358]) whilst pragmatists, a mechanism for monitoring quality, bias, and safety of AI prescriptions (68.4% [924/1350]).

The 2 psychological profile-types also have different expectations for the role played by hospitals in successful adoption of drug-prescribing AI. Regarding institutional culture, the majority of optimists emphasize that hospitals need to value innovation (80.3% [1090/1358]) and embrace new technology (76.0% [1032/1358]), whereas a higher proportion of (45.2% [610/1350]) pragmatists, compared with optimists (19.0% [258/1358]), emphasize that hospitals need to promote learning and talent development. To successfully implement drug-prescribing AI, the majority of optimists emphasize leadership of the hospital administration (72.2% [981/1358]), whilst pragmatists most emphasize the roles played by local AI “champions” (51.9% [701/1350]) and a multi-disciplinary task force (46.0% [621/1350]).

Optimists and pragmatists have different outlooks on their clinical practice after adopting drug-prescribing AI. Optimists, compared with pragmatists, are more likely to view drug-prescribing AI as an opportunity to enable them to focus on improving patient care throughput (66.9% [909/1358] vs. 25.0% [337/1350]; *P* < 0.0001), cost-effectiveness (63.0% [856/1358] vs. 31.3% [422/1350]; *P* < 0.0001), and empathetic caregiving (44.2% [600/1358] vs. 17.2% [232/1350]; *P* < 0.0001). In contrast, pragmatists stated they would emphasize the improvement of multi-disciplinary care (76.5% [1033/1350]), management of difficult-to-treat diseases (54.1% [730/1350]), and therapeutics research (44.5% [601/1350]) after adopting drug-prescribing AI.

Finally, the 2 psychological profile-types have different expectations for the government’s role in successful adoption of drug-prescribing AI. Optimists, compared with pragmatists, more emphasize the importance of a nation-wide policy mandate to promote AI in healthcare (*P* < 0.0001), whereas pragmatists, compared with optimists, more emphasize a nation-wide initiative for cultivating talents for medical AI (*P* < 0.0001). A higher proportion of optimists, compared with pragmatists, believe that professional medical societies and/or hospitals alone (that is, without involvement of regulatory authorities) could set the standards for drug-prescribing AI (38.9% [528/1358] vs. 23.6% [319/1350]; *P* < 0.0001). In contrast, the majority (54.2% [732/1350]) of the pragmatists indicated that standards should be set jointly by regulatory authorities, professional medical societies, and hospitals.

### Mechanisms underlying the genesis of psychological profile-types

Subsequently, we investigated potential mechanisms underlying the genesis of the 2 psychological profile-types.

**Fig. 3:**
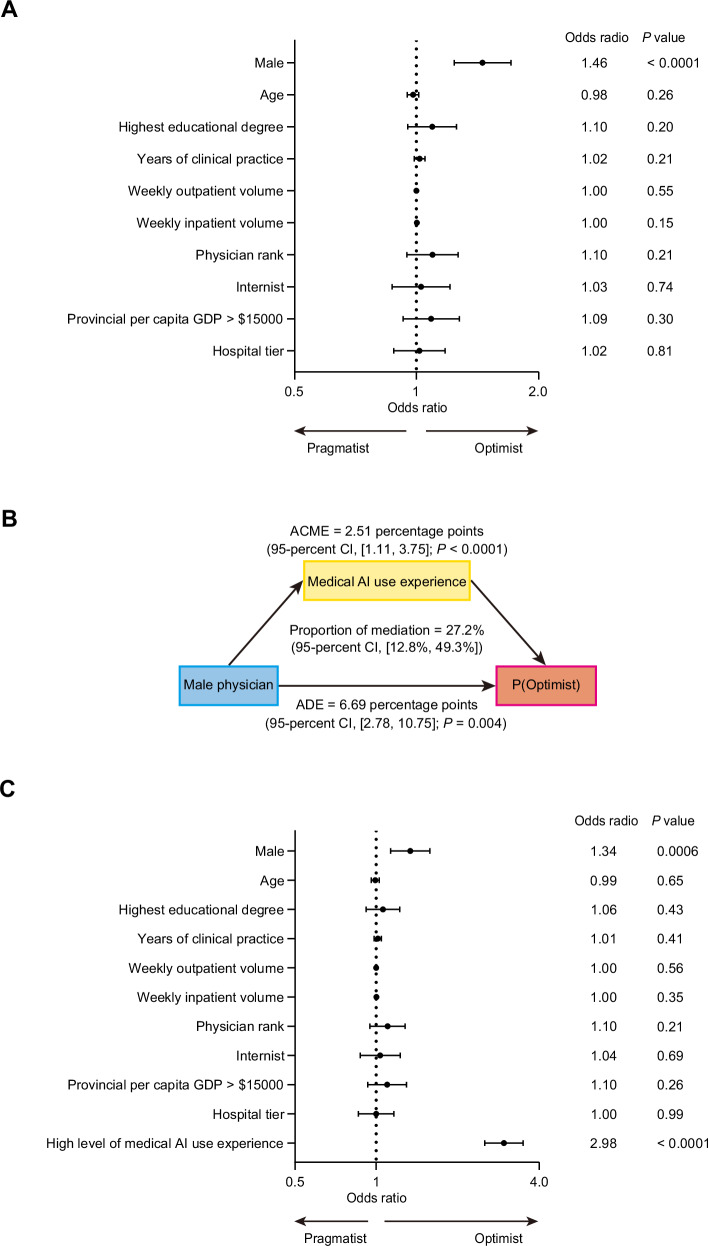
Determinants of Chinese physicians’ psychological profile-types with respect to drug-prescribing artificial intelligence (AI). **A** Effects of demographic co-variates and environmental factors (hospital tier and provincial per capita GDP) on psychological profile-type. Estimates and 95-percent confidence intervals (CIs) of odds ratios (ORs), calculated without bootstrapping, are displayed. GDP stands for gross domestic product and is expressed in US dollars ($). **B** The male sex’s effect on the probability of being an optimist mediated by medical AI use experience. P(Optimist) stands for the probability of being an optimist. ACME and ADE stand for average causal mediation effect and average direct effect, respectively. Displayed estimates and 95-percent CIs of ACME, ADE, and the proportion of mediation were calculated without bootstrapping. ACME and ADE passed significance (*P* < 0.05) in 98.3% (Supplementary Fig. 1) and 66.7% of 1000 independent bootstrapping experiments, respectively. **C** Independent effects of demographic co-variates, environmental factors, and self-assessed level of medical AI use experience on psychological profile-type. Estimates and 95-percent CIs of ORs, calculated without bootstrapping, are displayed. Independent effects of sex and level of medial AI use experiences passed significance in 66.5% and 98.9% (Supplementary Fig. 1) of 1000 bootstrapping experiments, respectively.

Multiple-variable logistic regression considering demographic co-variates (i.e., responses to Q1–Q6) and environmental factors, including hospital tier and provincial per capita GDP indicated that male physicians (OR = 1.46; 95-percent CI, [1.24, 1.72]; *P* < 0.0001) are more likely to be optimists compared with female physicians. The other co-variates including age, highest educational degree, clinical specialty, clinical practicing experience, physician rank, and provincial per capita GDP do not significantly contribute to the determination of psychological profile-type (Fig. 3A).

Causal mediation analysis indicated that the male sex’s total effect on the probability of being an optimist is 9.2 percentage points (95-percent CI, [4.9, 13.3]; *P* < 0.0001) compared with females, and 27.2% (95-percent CI, [12.8%, 49.3%]) of this total effect is mediated by the physician’s self-assessed level of experience using medical AI. Put otherwise, the average causal mediation effect (ACME) is 2.51 percentage points (95-percent CI, [1.11, 3.75]; *P* < 0.0001) increase in the probability of being an optimist (Fig. 3B). In contrast, physician rank is not a significant mediator of the male sex’s effect on the probability of being an optimist (ACME = –0.04 percentage points; 95-percent CI, [–0.30, 0.20]; *P* = 0.72) even though physician rank correlates with sex.

Finally, including medical AI use experience in multiple-variable logistic regression, we found that a high self-assessed level of using medical AI is associated with an OR of 2.98 (95-percent CI, [2.53, 3.51]; *P* < 0.0001) for being an optimist compared with physicians with a low level of use experience (Fig. 3C).

To assess the robustness of our findings, we ran 1000 independent bootstrapping experiments, each time re-sampling the 2708 respondents with replacement and repeating the clustering analysis, causal mediation analysis, and multiple-variable logistic regression. Our main results remained qualitatively the same (Supplementary Fig. 1). The male sex’s ACME via medical AI use experience passed significance (*P* < 0.05) in 98.3% of the bootstrapping experiments. In other words, medical AI use experience is a robust, significant mediator of sex’s effect on psychological profile-type. In contrast, sex’s average direct effect on psychological profile-type did not pass significance in 33.3% of the bootstrapping experiments. The independent effect of medical AI use experience on psychological profile-type is robust, passing significance in multiple-variable logistic regression in 98.9% of the bootstrapping experiments; the average OR for being optimists was 2.55 (SD = 0.66) in physicians with high vs. low self-assessed levels of medical use experience, after controlling for demographic co-variates and environmental factors.

## Discussion

The processes physicians use to prescribe drugs are poorly understood and subject to biases, heuristics, and extraneous influences with substantial intra- and inter-personal variation^12–15^. Even if physicians had access to all the multi-dimensional data needed to make the most rational or evidence-based drug prescription decision, they may not be able to review the data thoroughly because conscious thoughts have a limited bandwidth^16,17^. With AI assistance, physicians may be empowered to consider a much greater number of co-variates than they can currently process when they make a drug-prescribing decision^9^. However, prescribing drugs is an uncommonly studied area of AI research compared with other spheres of healthcare^4^. We also do not know whether and how physicians’ values and drug-prescribing decision-making processes might clash with AI’s^6^.

**Fig. 4:**
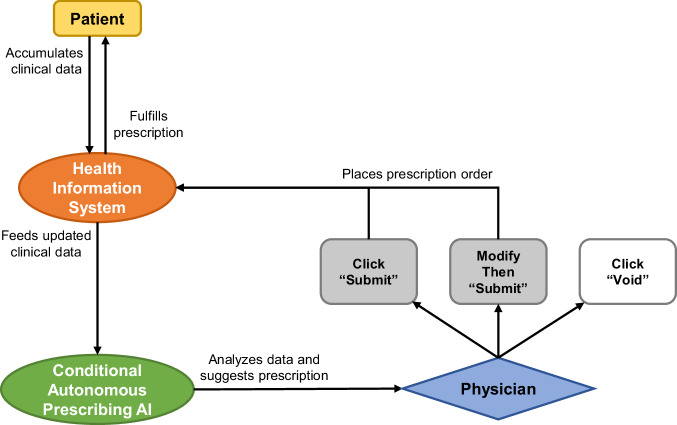
Proposed operational model for conditional autonomous drug-prescribing artificial intelligence (AI). Drug-prescribing AI (represented by the green oval) integrated into the health information system (orange oval) automatically monitors and analyzes patient data (yellow box), makes recommendation for drug prescription, and fills in all the details required for prescribing. The user interface is designed such that it is easy for physicians (blue diamond) to accept, modify, or veto the recommended prescription. The physicians' final decision is then automatically registered in the health information system.

There are two modes whereby AI could assist drug-prescribing decisions. One is fully autonomous AI without physician input or control. An alternative is conditional autonomous AI wherein AI automatically proposes a prescription which the physician can accept, modify, or veto (Fig. 4). With conditional autonomous drug-prescribing AI, the model must be integrated into the health information system so that AI automatically monitors and analyzes patient data, makes recommendation for drug prescription, and fills in all the details required for prescribing. Physicians can simply click the “Submit” button if they agree with the AI-driven prescription. If physicians reject the default prescription proposed by the AI, they can make changes to the AI-generated prescription and then click “Submit”, or they can veto the proposed prescription and click “Void”.

Several surveys have studied physicians’ attitudes towards medical AI^18–28^. However, most rely on convenience samples and do not study physicians’ attitudes towards AI-driven drug prescription. Our analysis of a large-sized, stratified sample indicates many Chinese physicians are receptive to using drug-prescribing AI and anticipate using it within 5 years. Chinese physicians think drug-prescribing AI would be most useful when there is a generally accepted treatment applicable to most patients with a particular medical condition (e.g., peri-operative medication management), when they are treating chronic illnesses, when many co-variates need to be considered to balance benefit and risk of prescribing a drug, or when complete reliance on humans may cause critical delay in intervention (e.g., continuous monitoring of cardiac arrhythmia). Most respondents indicated it is important a medical AI model, however effective, does not impinge on their professional autonomy, implying they prefer conditional to fully autonomous AI.

Most Chinese physicians do not believe drug-prescribing AI will supplant physician training. This seems reasonable: The more complex the clinical setting, the more necessary the requirement for physicians to have the competence to evaluate AI-generated prescription recommendations. In other words, medical AI in complex clinical settings is necessarily conditional, and the primary goal of conditional drug-prescribing AI is “complementing” physicians—i.e., empowering skilled physicians to care for more patients better—rather than “substituting” physicians^29^. With conditional AI, physicians maintain the authority on the final prescription decision in accordance with current regulations in most jurisdictions without negative impact on care quality, provided predeployment testing indicates that the average net effect of using AI is beneficial. However, there might be ethical and/or legal obligations to disclose the potential use of conditional drug-prescribing AI to patients who are members of subgroups vulnerable to algorithmic bias, especially if—in response to disclosure—the patients can realistically exercise agency to opt out of having their physicians utilize AI^30^.

**Fig. 5:**
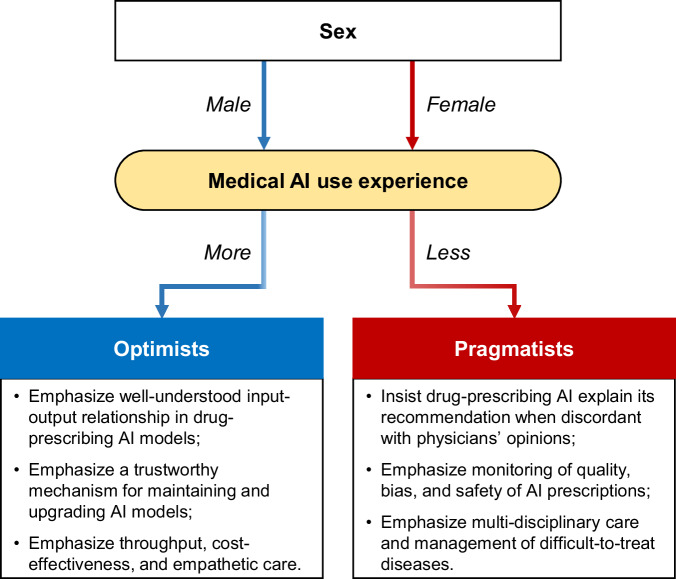
Proposed mechanisms underlying Chinese physicians’ receptiveness towards drug-prescribing artificial intelligence (AI). In China, male physicians (left) tend to have more experience using medical AI compared with female physicians (right). This sex disparity in medical AI use experience (yellow box) is a significant mediator that contributes to sex disparity in Chinese physicians’ optimism about drug-prescribing AI, resulting in that male physicians are more likely to be “optimists” (blue box) compared with females, whilst female physicians are more likely to be “pragmatists” (red box) compared with males.

The physicians we surveyed can be categorized into two types: optimists and pragmatists. Optimists emphasize throughput, cost-effectiveness, and development of a mechanism for maintaining and upgrading the model. Pragmatists, in contrast, insist an AI prescription model explain the bases for its recommendation—especially when it is discordant with their opinion—and there be monitoring of quality, bias, and safety of AI-driven drug prescriptions. Male physicians are more likely to be optimists compared with female physicians. However, sex is not a robust, direct cause of divergence in AI-optimism. Sex disparity in optimism about drug-prescribing AI is robustly and significantly mediated by disparity in experience with using medical AI between the sexes (Fig. 5). In fact, a high level of experience using medical AI is the strongest predictor for being an optimist.

Our finding has social equity implications. If optimism about AI is driven by prior exposure—which may be concentrated in better-resourced institutions—disparity among trainees of different institutions may become inadvertently entrenched if left without intervention. Graduates of low-resource medical schools may be resistant to AI technology because they had little prior exposure to AI, and their reserved attitude towards new technology might adversely affect their trajectory of professional development. On the other hand, graduates of better-resourced medical schools may be overly willing to put their trust in medical AI, a technology they perceive to be familiar and commonplace. Both under-utilization and overuse of medical AI have the potential to hurt patient welfare and safety. We propose that a core curriculum of medical AI practice and AI ethics should be instituted in all medical schools to uphold patient safety and promote workforce equity.

Contrary to what we had originally expected, age, educational level, physician rank, hospital tier, and provincial per capita GDP do not robustly influence physicians’ psychological profile-types, at least in China. It should be noted that the current study did not interrogate tier of medical school, physician’s income level, and other key sociodemographic and professional variables that may shape attitudes toward AI. The potential role played by these other factors needs to be studied.

Our study has limitations. First, it is China-focused, and our conclusions may not apply to other geographic regions. The majority of people in the emerging economies of Brazil, India, China, and South Africa report they are willing to trust AI; in contrast, people from other regions may not be as willing to trust AI^31^. Second, participant enrollment was biased towards tier-3 hospitals in the current study because, on average, a tier-3 hospital employs more physicians compared with a tier-2 or -1 hospital. Tier-3, -2, and -1 hospitals account for 52%, 23%, and 4% of hospital admissions in China^32^. Conclusions of the current study may not apply to primary healthcare physicians. Third, despite the study’s stratified sampling design, we cannot rule out potential selection bias. 23% of the participants failed to complete the survey or gave poor-quality responses. Because these people are plausibly less enthusiastic about AI compared with the other participants, our estimation for the percentage of optimists might be biased.

In conclusion, a high proportion of Chinese physicians are receptive to drug-prescribing AI, but they prefer conditional to fully autonomous AI. Initial settings for using drug-prescribing AI include situations where there is little ambiguity in a prescription and situations where prescribing involves navigating the enormous complexity of multi-dimensional clinical data. Enhancing development programs to improve exposure to medical AI may improve physicians’ optimism about drug-prescribing AI.

## Methods

### Ethical considerations

The survey study was approved by the Ethics Committee at the Institute of Hematology, Chinese Academy of Medical Sciences (QTJC2025047-EC-1) in accordance with the precepts of the Declaration of Helsinki. We had submitted the study protocol for peer-reviewed publication before enrolling the 1st subject^10^. All participants provided electronic written informed consent (*vide infra*).

### Sample size calculation

Setting type-I error *α* = 0.025 and type-II error *β* = 0.1, we estimated that at least 1515 physicians are needed to achieve a power of 90% to detect correlation *ρ*^2^ > 0.1 between physicians’ responses to survey questions—with up to 120 degrees of freedom—and physicians’ receptiveness towards drug-prescribing AI, assuming the true value of *ρ*^2^ is 0.16.

### Stratified sampling of survey participants

To ensure study participants better represent the physician population in China, the survey was not open or based on convenience samples. Rather, participants were recruited through stratified sampling.

First, 14 provincial-level administrative divisions (“provinces”) of China—Jiangsu, Fujian, Zhejiang, Tianjin, Inner Mongolia, Anhui, Liaoning, Sichuan, Tibet, Shanxi, Qinghai, Henan, Hebei, and Guangxi (listed in the order of their per capita gross domestic product [GDP] in 2024 [from high to low] according to data from the PRC National Bureau of Statistics^11^)—were selected, representing a broad range of economic development levels.

Subsequently, 76 hospitals, including 32 tier-3 (*sanji*; highest-level), 25 tier-2 (*erji*), and 19 tier-1 (*yiji*; lowest-level) hospitals, were selected from the 14 provinces (Supplementary Table 1). A tier-1 hospital is a community clinical service center, whilst a tier-2 hospital is a mid-sized hospital, and a tier-3 hospital, a large-sized hospital or an academic medical center. Hospitals were not selected randomly from the set of all existing hospitals because there is wide variation of patient volume among hospitals. Rather, for each province, we first selected 1–6 tier-3 hospitals well-recognized in their respective regions and then charged the participating tier-3 hospitals with the task of inviting local tier-2 and -1 hospitals that they partner with and that have higher patient volume to participate in the study. Therefore, our survey study was biased towards higher-impact hospitals, whilst clinics at remote, less-populated areas were underrepresented. 13 surveyed hospitals are “Chinese medicine hospitals” or “integrated Chinese and western medicine hospitals” that include western medicine departments.

At each surveyed institution, its hospital administration invited a sample of staff internists and surgeons to participate in the survey. The hospital administration was instructed to ensure that participants were a balanced representation of 2 clinical specialties—internal medicine and surgery—and 3 successive ranks in the professional ladder: “chief physician” (*zhuren yishi*; highest-rank), “associate chief physician” (*fuzhuren yishi*), and “junior attending physician” (*zhuzhi yishi*; lowest-rank). Participation was voluntary and anonymous. No incentive was offered to the survey participants.

### Survey administration and electronic written informed consent

Once the hospital administration at a surveyed institution decided on a date for survey delivery, the central research team in Tianjin dispatched researchers to be physically present at the survey-taking session to serve as moderators, whose primary responsibility was to ensure that there was no technical error or misunderstanding of survey questions. Each separate survey-taking session had its own separate survey web page hosted on secure Alibaba Cloud servers and accessible via a unique Quick Response (QR) code. The QR code had a preset alive period and expired once the survey-taking session ended. Participants scanned the QR code with smartphones to access the survey web page. Once a participant connected to the survey web page, the message “I understand the purpose of this survey and agree to participate.” was displayed on the phone screen, and the participant was prompted to enter his/her mobile phone number if he/she agreed to participate. A unique verification code was then sent to the participant’s smartphone via short message service (SMS), and to confirm his/her consent to participate in the survey, the participant sent the verification code back, thereby providing his/her e-signature. Each phone number could be used only once for survey-taking. Entered phone numbers were stored in password-protected secure Alibaba Cloud servers. Researchers were forbidden from contacting the participants using any means after the survey-taking session ended.

### Pilot study

Prior to finalization of survey questions, a draft questionnaire was tested on 20 physicians at the Institute of Hematology, Chinese Academy of Medical Sciences (Tianjin, China) and modified and improved based on their feedback. Subsequently, the survey was tested at 1 tier-3 and 2 tier-2 hospitals in Tibet. This pilot study allowed us to fine-tune the logistics of survey delivery before rolling out the survey to other provinces. We did not modify the contents of survey questions after the pilot study conducted in Tibet.

### Data collection

Survey delivery was via smartphones. The survey consisted of 32 questions (Supplementary Table 2), interrogating the physician’s demographic information (Q1–Q6), self-assessed levels of AI-related knowledge and use experience (Q7–Q10), outlook on the future of AI in healthcare (Q11–Q15), trade-off between physicians’ professional autonomy and the efficacy of an AI model (Q16), projected initial settings of using drug-prescribing AI (Q17), perceived importance of 5 technological attributes of drug-prescribing AI models (Q18, vetted efficacy; Q19, expediency; Q20, transparency; Q21, explainability; Q22, governance/stewardship; Q23, ranking of the relative importance of the 5 technological attributes), perceived importance of hospital attributes in successful adoption of drug-prescribing AI (Q24, institutional culture; Q25, change management; Q26, information technology [IT] and data science; Q27, differentiation and future opportunities), perceived importance of governmental attributes in successful adoption of drug-prescribing AI (Q28, nation-wide commitments; Q29, AI standards; Q30, remuneration policy), and 2 final questions regarding self-assessed readiness for adopting drug-prescribing AI (Q31 and Q32)^10^.

Q7, Q8, Q9, Q10, Q11, Q12, Q13, Q14, Q15, and Q20 were Likert-scale questions with explanatory wordings added to calibrate participants’ interpretation of the 5 option items: “Strongly Disagree”, “Disagree”, “Neutral”, “Agree”, and “Strongly Agree” (Supplementary Table 2). For example, when inquiring whether a participant is experienced with using medical AI, Q10 specified that “Strongly Agree” means “NOT ONLY am I good at using medical AI BUT ALSO I constantly and actively learn new skills to expand my toolbox for medical AI.”

Q16, Q17, Q18, Q19, Q21, Q22, Q23, Q24, Q25, Q26, Q27, and Q28 were forced-choice questions (Supplementary Table 2). For example, Q16 requested each participant to decide which one of the 2 options is more important to him/her: “A medical AI system is efficacious in delivering what it promises” vs. “A medical AI system does not affect my autonomy as a physician”.

Each page displayed one question only. Participants were able to switch between questions and modify their answers as long as they have not clicked “Final Submission”. Items in multiple-choice questions were not randomized, and we did not scramble the order of the questions for different participants, because many questions were Likert-scale questions and there was a natural progression of the themes asked by the questions. Survey was not adaptive; that is, each respondent was expected to answer all the 32 questions.

Proper answering of survey questions was enforced by on-the-fly data quality checks. For example, if a respondent chose >3 option items in Q19, which nevertheless stipulated he/she could choose only ≤3, the respondent would not be able to submit his/her response to Q19. A participant’s responses to survey questions were stored sequentially and cumulatively, and if he/she accidentally exited the survey before completing it, he/she could re-enter the survey using his/her mobile phone number (with SMS code verification), and his/her prior data entries would be automatically restored. Participants could not click “Final Submission” unless all the survey questions have been answered. Once “Final submission” was clicked, participants could not re-enter the survey to modify their answers. All entered data are stored in password-protected, secure Alibaba Cloud servers.

### Post-collection data quality control

Completion rate was calculated by the number of participants who clicked “Final Submission” divided by the total number of participants who initiated survey-taking. Questionnaires that were filled out in < 3 min or failed to pass basic data-quality checks were classified as “bad responses” and excluded from analyses. Basic data-quality checks included: (1) starting clinical practice ≥ age 21; (2) weekly outpatient volume ≤ 1225 persons; (3) weekly inpatient volume ≤ 120 persons; and (4) logical coherence between responses to Q9 and Q10 and between responses to Q31 and Q32. (If a participant selected “Disagree” or “Strongly Disagree” in Q9, his/her response to Q10 should also be “Disagree” or “Strongly Disagree”. Response to Q31 should not be more pessimistic than response to Q32.)

### Analyses

Responses to all survey questions were converted to numerical values before analyses. Responses to Likert-scale questions were converted to 5 (Strongly Agree) to 1 (Strongly Disagree). For a forced-choice question stipulating that the participant should select no more than $n$ items, the 1st-, 2nd-, …, and nth-priority choices were coded as $n$, $n-1$,…, and $1$, respectively, and unselected items were coded as $0$. Responses to Q31 and Q32 were coded as follows: “≤ 1 year”, 6; “1.1 to 3 years”, 5; “3.1 to 5 years”, 4; “5.1 to 10 years”, 3; “> 10 years”, 2; and “Never”, 1.

Physicians’ demographic co-variates (Q1–Q6) and levels of AI-related knowledge and use experience (Q7–Q10) were treated as the physicians’ basic attributes whilst hospital tier and provincial per capita GDP, the environmental factors. Responses to Q11–Q32 were interpreted as physicians’ psychological characteristics.

Hierarchical clustering (using the correlation metric and the complete-linkage criterion) of the respondents was performed based on their psychological characteristics (i.e., Q11–Q32) to uncover their psychological profile-types. Because respondents might vary in how they interpreted the Likert scales in the questionnaire (e.g., the full range “Strongly Agree–Strongly Disagree” might shrink to “Agree–Disagree” in responses of participants who were moderate in their responses), we used the correlation metric, which measures the similarity of response patterns, as the distance metric. The complete-linkage criterion, which merges groups based on the largest distance over all possible pairs, was used because it does not assume that responses can be averaged across participants (as in the average-linkage criterion) or that the distance metric is Euclidean (as in Ward’s linkage).

We used 2-sided Wilcoxon test (for cardinal or ordinal data) and chi-squared test (for distributions) to compare the 2 profile-types’ psychological characteristics. Statistical significance was defined as unadjusted *P* < 0.0001. The cut-off for *P* value was set to be stringent to account for multiple-hypotheses testing. All reported *P* values are unadjusted.

We used multiple-variable logistic regression to estimate how psychological profile-types are determined by demographic co-variates, hospital tier, and provincial per capita GDP: OPTIMIST ~ MALE + AGE + HIGHEST_EDUCATIONAL_DEGREE + YEARS_OF_CLINICAL_PRACTICE + WEEKLY_OUTPATIENT_VOLUME + WEEKLY_INPATIENT_VOLUME + PHYSICIAN_RANK + INTERNIST + PROVINCIAL_PER_CAPITA_GDP_OVER_USD15000 + HOSPITAL_TIER. (OPTIMIST = 1 and 0 for optimists and pragmatists, respectively. MALE = 1 and 0 for males and females, respectively. HIGHEST_EDUCATIONAL_DEGREE = 3, 2, 1, and 0 for doctorate, master, bachelor of medicine, and associate, respectively. PHYSICIAN_RANK = 2, 1, and 0 for chief physicians, associate chief physicians, and junior attending physicians, respectively. INTERNIST = 1 and 0 for internists and surgeons, respectively. PROVINCIAL_PER_CAPITA_GDP_OVER_USD15000 = 1 if provincial per capita GDP was > $15,000 in 2024 according to data from the PRC National Bureau of Statistics^11^; otherwise, its value was set to be 0. HOSPITAL_TIER = 3, 2, and 1 for tier-3, -2, and -1 hospitals, respectively.) Statistical significance was defined as unadjusted *P* < 0.05. All reported *P* values are unadjusted.

To interrogate whether the effect of sex on psychological profile-type was mediated by the physician’s self-assessed level of experience using medical AI, we conducted causal mediation analysis using the R function “mediate” in the package “mediation” (version 4.5.1)^33^. The treatment variable was MALE. The mediator variable was MEDICAL_AI_USE_EXPERIENCE. (MEDICAL_AI_USE_EXPERIENCE = 1 if response to Q10 was “Agree” or “Strongly Agree”; otherwise, MEDICAL_AI_USE_EXPERIENCE = 0.) The mediator model was the logistic regression model: MEDICAL_AI_USE_EXPERIENCE ~ MALE + AGE + HIGHEST_EDUCATIONAL_DEGREE + YEARS_OF_CLINICAL_PRACTICE + WEEKLY_OUTPATIENT_VOLUME + WEEKLY_INPATIENT_VOLUME + PHYSICIAN_RANK + INTERNIST + PROVINCIAL_PER_CAPITA_GDP_OVER_USD15000 + HOSPITAL_TIER. The output model was the logistic regression model: OPTIMIST ~ MEDICAL_AI_USE_EXPERIENCE + MALE + AGE + HIGHEST_EDUCATIONAL_DEGREE + YEARS_OF_CLINICAL_PRACTICE + WEEKLY_OUTPATIENT_VOLUME + WEEKLY_INPATIENT_VOLUME + PHYSICIAN_RANK + INTERNIST + PROVINCIAL_PER_CAPITA_GDP_OVER_USD15000 + HOSPITAL_TIER. We calculated estimates and 95-percent confidence intervals of the average causal mediation effect (ACME), the average direct effect (ADE), and the proportion of mediation. Statistical significance was defined as unadjusted *P* < 0.05. All reported *P* values are unadjusted.

We also interrogated whether the effect of sex on psychological profile-type is mediated by physician rank: the treatment variable was MALE, and the mediator variable was CHIEF_PHYSICIAN. (CHIEF_PHYSICIAN = 1 and 0 for chief physicians and other physician ranks, respectively.) The mediator model was the logistic regression model: CHIEF_PHYSICIAN ~ MALE + AGE + HIGHEST_EDUCATIONAL_DEGREE + YEARS_OF_CLINICAL_PRACTICE + WEEKLY_OUTPATIENT_VOLUME + WEEKLY_INPATIENT_VOLUME + INTERNIST + PROVINCIAL_PER_CAPITA_GDP_OVER_USD15000 + HOSPITAL_TIER. The output model was the logistic regression model: OPTIMIST ~ CHIEF_PHYSICIAN + MALE + AGE + HIGHEST_EDUCATIONAL_DEGREE + YEARS_OF_CLINICAL_PRACTICE + WEEKLY_OUTPATIENT_VOLUME + WEEKLY_INPATIENT_VOLUME + INTERNIST + PROVINCIAL_PER_CAPITA_GDP_OVER_USD15000 + HOSPITAL_TIER. Statistical significance was defined as unadjusted *P* < 0.05.

Finally, to assess the robustness of our results, we ran 1000 independent bootstrapping experiments. In each bootstrapping experiment, on average $1-\exp \left(-1\right)=63 \%$ of the participants were randomly selected (with possible duplicates) to be included in the bootstrap sample. We performed hierarchical clustering on the bootstrap sample and subsequently repeated the mediation analyses and multiple-variable logistic regression. We calculated the average estimates and standard deviations of the male sex’s ADE and ACME—via self-assessed level of experience with using medical AI—on the probability of being an optimist and of the odds ratio (OR) for being an optimist in physicians with high vs. low levels of medical AI use experience (controlling for demographic co-variates, hospital tier, and provincial per capita GDP) and how many times the ADE, ACME, and OR passed significance (unadjusted *P* < 0.05) in the bootstrapping experiments.

## Supplementary information

Supplementary information

## Data Availability

Anonymized data are available upon reasonable request addressed to Junren Chen.

## Code availability

Computational codes are available upon reasonable request addressed to Junren Chen.
