# Supplementary material for: Receptiveness of physicians towards artificial intelligence-driven drug prescription: a nationwide survey
Source: Npj Health Syst. 2026 Jun 8;3:44. doi: 10.1038/s44401-026-00101-3 (PMC13354231; doi:10.1038/s44401-026-00101-3)
Supplement: Supplementary file 1 — Supplementary information [file 44401_2026_101_MOESM1_ESM.docx]

**Supplement Table 1. Surveyed hospitals.**

| **Provincial-level administrative division** | **Hospital name (English)** | **Hospital name (Chinese)** | **Hospital tier** | **Number of participants** | **Number of respondents passing data quality checks** |
| --- | --- | --- | --- | --- | --- |
| Anhui | Anhui Campus of the Second Affiliated Hospital, Zhejiang University School of Medicine; The First Affiliated Hospital of Bengbu Medical College | 安徽省蚌埠医科大学第一附属医院 | Tier-3 | 71 | 58 |
| Anhui | Feidong County People's Hospital, Hefei City | 安徽省合肥市肥东县人民医院 | Tier-3 | 74 | 54 |
| Anhui | Jinzhai County Hospital of Traditional Chinese Medicine, Liuan City | 安徽省六安市金寨县中医医院 | Tier-3 | 117 | 86 |
| Anhui | The First Affiliated Hospital of Anhui University of Chinese Medicine | 安徽中医药大学第一附属医院 | Tier-3 | 79 | 67 |
| Anhui | The Second Affiliated Hospital of Bengbu Medical University | 安徽省蚌埠医科大学第二附属医院 | Tier-3 | 8 | 7 |
| Anhui | Xuancheng People's Hospital | 安徽省宣城市人民医院 | Tier-3 | 64 | 49 |
| Anhui | Hospital Affiliated With Huaiyuan County Health School | 安徽省蚌埠市怀远县卫生学校附属医院 | Tier-1 | 11 | 8 |
| Fujian | Second Affiliated People's Hospital of Fujian University of Traditional Chinese Medicine | 福建中医药大学附属第二人民医院 | Tier-3 | 16 | 15 |
| Fujian | The Second Affiliated Hospital of Fujian Medical University | 福建医科大学第二附属医院 | Tier-3 | 110 | 82 |
| Fujian | Quanzhou Municipal Dongnan Hospital | 福建省泉州市东南医院 | Tier-2 | 34 | 31 |
| Fujian | Quanzhou Xingxian Hospital, Licheng District, Quanzhou City | 福建省泉州市鲤城区兴贤医院 | Tier-2 | 15 | 12 |
| Fujian | Kaiyuan Subdistrict Community Hospital, Licheng District, Quanzhou City | 福建省泉州市鲤城区开元街道社区卫生服务中心 | Tier-1 | 15 | 14 |
| Guangxi | The First Affiliated Hospital of Guangxi University of Chinese Medicine | 广西中医药大学第一附属医院 | Tier-3 | 97 | 73 |
| Guangxi | Liuzhou City Hospital of Integrated Chinese and Western Medicine | 广西柳州市中西医结合医院 | Tier-2 | 14 | 11 |
| Guangxi | Min Sheng Ning Hospital, Dahua District, Hechi City | 广西河池市大化县民生宁医院 | Tier-2 | 3 | 1 |
| Guangxi | People's Hospital of Yongning District, Nanning City | 广西南宁市邕宁区人民医院 | Tier-2 | 8 | 6 |
| Guangxi | Guibin Hospital, Binyang County, Nanning City | 广西南宁市宾阳桂宾医院 | Tier-1 | 45 | 30 |
| Guangxi | Pingshan Township Hospital, Luzhai County, Liuzhou City | 广西柳州市鹿寨县平山镇卫生院 | Tier-1 | 6 | 4 |
| Guangxi | Tanzhong Community Hospital, Chengzhong District, Liuzhou City | 广西柳州市城中区潭中社区卫生院服务中心 | Tier-1 | 14 | 10 |
| Hebei | The Second Hospital of Hebei Medical University | 河北医科大学第二医院 | Tier-3 | 101 | 70 |
| Hebei | Central Hospital of Qinghe County, Xingtai City | 河北省邢台市清河县中心医院 | Tier-2 | 42 | 34 |
| Hebei | Hospital of fengnan District, Tangshan | 河北省唐山市丰南区医院 | Tier-2 | 12 | 10 |
| Hebei | Huanggezhuang Township Central Hospital, Fengnan District, Tangshan City | 河北省唐山市丰南区黄各庄镇中心卫生院 | Tier-1 | 17 | 10 |
| Hebei | Lianzhuang Central Hospital, Qinghe County, Xingtai City | 河北省邢台市清河县连庄中心卫生院 | Tier-1 | 22 | 13 |
| Henan | Central Hospital of Kaifeng | 河南省开封市中心医院 | Tier-3 | 85 | 62 |
| Henan | The Second Hospital of Traditional Chinese Medicine of Kaifeng | 河南省开封市第二中医院 | Tier-3 | 107 | 96 |
| Henan | Qi County People's Hospital, Kaifeng City | 河南省开封市杞县中心医院 | Tier-2 | 21 | 15 |
| Henan | Tongxu County Central Hospital, Kaifeng City | 河南省开封市通许县中心医院 | Tier-2 | 42 | 30 |
| Henan | Gongye Subdistrict Community Hospital, Kaifeng City | 河南省开封市工业社区卫生服务中心 | Tier-1 | 5 | 5 |
| Henan | Xinghuaying Township Hospital, Kaifeng City | 河南省开封市城乡一体化示范区杏花营镇卫生院 | Tier-1 | 16 | 14 |
| Inner Mongolia | Inner Mongolia Autonomous Region Hospital of Traditional Chinese Medicine | 内蒙古自治区中医院 | Tier-3 | 92 | 72 |
| Inner Mongolia | The Affiliated Hospital of Inner Mongolia Medical University | 内蒙古医科大学附属医院 | Tier-3 | 70 | 55 |
| Inner Mongolia | Helingeer County People's Hospital, Hohhot City | 内蒙古呼和浩特市和林格尔县人民医院 | Tier-2 | 32 | 26 |
| Inner Mongolia | Hohhot Xincheng Hospital | 内蒙古呼和浩特市新城区医院 | Tier-2 | 32 | 24 |
| Inner Mongolia | Xinghuaxiang Community Hospital, Hohhot City | 内蒙古呼市新城区西街办事处星火巷社区卫生服务站 | Tier-1 | 4 | 3 |
| Inner Mongolia | Xingongzhong Township Hospital, Wuyuan County, Bayannur City | 内蒙古巴彦淖尔市五原县新公中镇卫生院 | Tier-1 | 15 | 10 |
| Jiangsu | The Second Affiliated Hospital of Xuzhou Medical University | 江苏省徐州矿务集团总医院 | Tier-3 | 108 | 84 |
| Jiangsu | The Third Hospital of Xuzhou Kuangwujituan | 江苏省徐州矿务集团第三医院 | Tier-2 | 23 | 17 |
| Liaoning | Liaoning Electric Power Center Hospital | 辽宁电力中心医院 | Tier-3 | 73 | 60 |
| Liaoning | Liaoning University of Traditional Chinese Medicine Hospital subsidiary Fourth | 辽宁中医药大学附属第四医院 | Tier-3 | 115 | 94 |
| Liaoning | Hunnan District Hospital, Shenyang City | 辽宁省沈阳市浑南区医院 | Tier-2 | 17 | 13 |
| Liaoning | Liaozhong District People's Hospital, Shenyang City | 辽宁省沈阳市辽中区人民医院 | Tier-2 | 38 | 30 |
| Liaoning | The Ninth People's Hospital of Shenyang | 辽宁省沈阳市第九人民医院 | Tier-2 | 37 | 32 |
| Liaoning | Liaozhong District Hospital of Traditional Chinese Medicine, Shengyang City | 辽宁省沈阳市辽中区中医院 | Tier-1 | 17 | 13 |
| Qinghai | Huzhu Tu Ethnicity Autonomous County People's Hospital, Haidong City | 青海省海东市互助土族自治县人民医院 | Tier-3 | 85 | 72 |
| Qinghai | Qinghai Provincial Hospital of Traditional Chinese Medicine | 青海省中医院 | Tier-3 | 16 | 14 |
| Qinghai | Qinghai Red Cross Hospital | 青海红十字医院 | Tier-3 | 173 | 121 |
| Qinghai | The Fifth People's Hospital of Qinghai Province | 青海省第五人民医院 | Tier-3 | 36 | 28 |
| Qinghai | The First People's Hospital of Haibei Tibetan Autonomous Prefecture | 青海海北藏族自治州第一人民医院 | Tier-2 | 19 | 13 |
| Qinghai | The First People's Hospital of Haidong City | 青海海东市第一人民医院 | Tier-2 | 35 | 32 |
| Shanxi | Second Hospital of Shanxi Medical University | 山西医科大学第二医院 | Tier-3 | 63 | 49 |
| Shanxi | Sinopharm Tongmei Central Hospital, Datong City | 山西省大同市国药同煤总医院 | Tier-3 | 113 | 90 |
| Shanxi | Sinopharm Tongmei Second Hospital, Datong City | 山西省大同市国药同煤二医院 | Tier-2 | 31 | 24 |
| Shanxi | The Second People's Hospital of Datong Cancer Hospital | 山西省大同市第二人民医院 肿瘤医院 | Tier-2 | 16 | 14 |
| Shanxi | Pingquan Community Hospital, Yungang District, Datong City | 山西省大同市云冈区平泉社区卫生服务中心 | Tier-1 | 6 | 4 |
| Shanxi | Tairongli Community Hospital, Heshun Subdistrict, Yungang District, Datong City | 山西省大同市云冈区和顺街道泰荣里社区服务中心 | Tier-1 | 8 | 2 |
| Sichuan | Chinese Medicine Hospital of Zhongjiang, Deyang City | 四川省德阳市中江县中医医院 | Tier-3 | 13 | 11 |
| Sichuan | Deyang People's Hospital | 四川省德阳市人民医院 | Tier-3 | 82 | 70 |
| Sichuan | West China Hospital of Sichuan University | 四川大学华西医院 | Tier-3 | 8 | 8 |
| Sichuan | The Six People's Hospital of Deyang City | 四川省德阳市第六人民医院 | Tier-2 | 31 | 26 |
| Sichuan | Bajiao Community Health Service Center, Jingyang District, Deyang City | 四川省德阳市旌阳区八角社区卫生服务中心 | Tier-1 | 4 | 2 |
| Tianjin | Hospital of Integrated Chinese and Western Medicine, Tianjin | 天津市中西医结合医院 | Tier-3 | 136 | 94 |
| Tianjin | Tianjin First Central Hospital | 天津市第一中心医院 | Tier-3 | 50 | 33 |
| Tianjin | Tianjin Jinghai District Hospital | 天津市静海区医院 | Tier-3 | 203 | 154 |
| Tianjin | Huang He Hospital | 天津市黄河医院(天津市体育医院) | Tier-2 | 41 | 37 |
| Tianjin | Daqiuzhuang Township Central Hospital, Jinghai District, Tianjin | 天津市静海区大邱庄镇中心卫生院 | Tier-1 | 12 | 12 |
| Tianjin | Tangguantun Township Central Hospital, Jinghai District, Tianjin | 天津市静海区唐官屯镇中心卫生院 | Tier-1 | 9 | 7 |
| Tibet | Xizang Autonomous Region People’s Hospital | 西藏自治区人民医院 | Tier-3 | 81 | 67 |
| Tibet | Duilong Deqing District People’s Hospital, Lhasa City | 西藏拉萨市堆龙德庆区人民医院 | Tier-2 | 16 | 13 |
| Tibet | Xizang Autonomous Region Judicial Police Hospital | 西藏自治区司法警官医院 | Tier-2 | 6 | 3 |
| Zhejiang | Huzhou Central Hospital | 浙江省湖州市中心医院 | Tier-3 | 99 | 80 |
| Zhejiang | Huzhou Hospital of Traditional Chinese Medicine Affiliated to Zhejiang University of Traditional Chinese Medicine | 浙江省湖州市中医院 | Tier-3 | 87 | 65 |
| Zhejiang | Nanxun People‘s Hospital, Huzhou City | 浙江省湖州市南浔区人民医院 | Tier-2 | 37 | 31 |
| Zhejiang | Wuxing People's Hospital, Huzhou City | 浙江省湖州市吴兴区人民医院 | Tier-2 | 12 | 5 |
| Zhejiang | Jiuguan Subdistrict Community Hospital, Nanxun District, Huzhou City | 浙江省湖州市南浔区旧馆街道社区卫生服务中心 | Tier-1 | 7 | 6 |
| Zhejiang | Zhili Township Hospital, Wuxing District, Huzhou City | 浙江省湖州市吴兴区织里镇卫生院 | Tier-1 | 14 | 11 |

**Supplement Table 2. Characteristics and responses of surveyed physicians.**

| **Attribute/Response** | **All**  (n = 2708) | **Optimists**  (n = 1358) | **Pragmatists**  (n = 1350) | ***P* value** (Optimists *vs.* Pragmatists) |
| --- | --- | --- | --- | --- |
| **Provincial-level administrative division** |  |  |  | 0.15 ^a^ |
| Anhui | 329 (12.1%) | 163 (12.0%) | 166 (12.3%) |  |
| Fujian | 154 (5.7%) | 90 (6.6%) | 64 (4.7%) |  |
| Guangxi | 135 (5%) | 69 (5.1%) | 66 (4.9%) |  |
| Hebei | 137 (5.1%) | 76 (5.6%) | 61 (4.5%) |  |
| Henan | 222 (8.2%) | 111 (8.2%) | 111 (8.2%) |  |
| Inner Mongolia | 190 (7%) | 93 (6.8%) | 97 (7.2%) |  |
| Jiangsu | 101 (3.7%) | 58 (4.3%) | 43 (3.2%) |  |
| Liaoning | 242 (8.9%) | 105 (7.7%) | 137 (10.1%) |  |
| Qinghai | 280 (10.3%) | 149 (11.0%) | 131 (9.7%) |  |
| Shanxi | 183 (6.8%) | 86 (6.3%) | 97 (7.2%) |  |
| Sichuan | 117 (4.3%) | 52 (3.8%) | 65 (4.8%) |  |
| Tianjin | 337 (12.4%) | 173 (12.7%) | 164 (12.1%) |  |
| Tibet | 83 (3.1%) | 35 (2.6%) | 48 (3.6%) |  |
| Zhejiang | 198 (7.3%) | 98 (7.2%) | 100 (7.4%) |  |
| **Type of hospital** ^‡^ |  |  |  | 0.17 ^a^ |
| Tier-3 hospital | 2040 (75.3%) | 1042 (76.7%) | 998 (73.9%) |  |
| Tier-2 hospital | 490 (18.1%) | 227 (16.7%) | 263 (19.5%) |  |
| Tier-1 hospital | 178 (6.6%) | 89 (6.6%) | 89 (6.6%) |  |
| **Q1. Age** | 40 (35 – 46) ^†^ | 40 (35 – 47) ^†^ | 39 (35 – 45) ^†^ | 0.04 ^b^ |
| **Q2. Sex** |  |  |  | <0.0001 ^a^ |
| Male | 1301 (48%) | 719 (52.9%) | 582 (43.1%) |  |
| Female | 1407 (52%) | 639 (47.1%) | 768 (56.9%) |  |
| **Q3. Highest educational degree** |  |  |  | 0.11 ^b^ |
| Associate | 93 (3.4%) | 50 (3.7%) | 43 (3.2%) |  |
| Bachelor of medicine | 1420 (52.4%) | 689 (50.7%) | 731 (54.1%) |  |
| Master | 1037 (38.3%) | 528 (38.9%) | 509 (37.7%) |  |
| Doctorate | 158 (5.8%) | 91 (6.7%) | 67 (5.0%) |  |
| **Q4.** I started practicing clinical medicine at age __. | 25 (23 – 27) ^†^ | 25 (23 – 27) ^†^ | 25 (24 – 27) ^†^ | 0.12 ^b^ |
| On average I see ≈__ outpatients in a week. | 40 (20 – 80) ^†^ | 40 (20 – 80) ^†^ | 31 (20 – 80) ^†^ | 0.11 ^b^ |
| On average I am involved in management of ≈__ inpatients in a week. | 12 (7 – 20) ^†^ | 13 (8 – 20) ^†^ | 10 (6 – 20) ^†^ | 0.0010 ^b^ |
| **Q5. Current physician rank** |  |  |  | 0.006 ^b^ |
| Chief physician (*zhuren yishi*) | 383 (14.1%) | 228 (16.8%) | 155 (11.5%) |  |
| Associate chief physician (*fuzhuren yishi*) | 762 (28.1%) | 371 (27.3%) | 391 (29.0%) |  |
| Junior attending physician (*zhuzhi yishi*) | 1563 (57.7%) | 759 (55.9%) | 804 (59.6%) |  |
| **Q6. Clinical specialty** |  |  |  | 0.24 ^a^ |
| Internal medicine | 1640 (60.6%) | 807 (59.4%) | 833 (61.7%) |  |
| Surgery | 1068 (39.4%) | 551 (40.6%) | 517 (38.3%) |  |
| **Q7. I am knowledgeable in artificial intelligence (AI) technology in general (that is, not limited to application of AI in medicine)** |  |  |  | <0.0001 ^b^ |
| Strongly agree *I am confident to make decisions regarding AI technology based on my own judgment.* | 451 (16.7%) | 311 (22.9%) | 140 (10.4%) |  |
| Agree *I can make my own decisions regarding AI technology after consulting with experts and/or perusing literature.* | 1043 (38.5%) | 585 (43.1%) | 458 (33.9%) |  |
| Neutral  *If there are experts showing me options for AI technology and explaining to me their pros and cons, I am confident I will make the best technological decision based on the presented options.* | 1167 (43.1%) | 445 (32.8%) | 722 (53.5%) |  |
| Disagree *I am aware of technological trends of AI but I would rather someone else make decisions for me regarding AI technology.* | 38 (1.4%) | 16 (1.2%) | 22 (1.6%) |  |
| Strongly disagree  *I am unfamiliar with technological trends of AI and would rather someone else make decisions for me regarding AI technology.* | 9 (0.3%) | 1 (0.1%) | 8 (0.6%) |  |
| **Q8. I am knowledgeable in medical AI technology** |  |  |  | <0.0001 ^b^ |
| Strongly agree *I am confident to make decisions regarding the use of medical AI technology based on my own judgment.* | 375 (13.8%) | 269 (19.8%) | 106 (7.9%) |  |
| Agree *I can make my own decisions regarding the use of medical AI technology after consulting with experts and/or perusing literature.* | 1041 (38.4%) | 593 (43.7%) | 448 (33.2%) |  |
| Neutral *If there are experts showing me options for medical AI technology and explaining to me their pros and cons, I am confident I will make the best technological decision for medical AI based on the presented options.* | 1223 (45.2%) | 473 (34.8%) | 750 (55.6%) |  |
| Disagree  *I am aware of technological trends of medical AI but I would rather someone else make decisions for me regarding medical AI technology.* | 56 (2.1%) | 20 (1.5%) | 36 (2.7%) |  |
| Strongly disagree  *I am unfamiliar with technological trends of medical AI and would rather someone else make decisions for me regarding medical AI technology.* | 13 (0.5%) | 3 (0.2%) | 10 (0.7%) |  |
| **Q9. I am experienced with using AI in work and/or daily life (that is, not limited to application of AI in medicine)** |  |  |  | <0.0001 ^b^ |
| Strongly agree  *NOT ONLY am I good at using AI in my work and/or daily life BUT ALSO I constantly and actively learn new skills to expand my AI toolbox.* | 408 (15.1%) | 290 (21.4%) | 118 (8.7%) |  |
| Agree *I use AI in my work and/or daily life AND I am good at using AI tool(s).* | 1000 (36.9%) | 568 (41.8%) | 432 (32.0%) |  |
| Neutral  *I have some experience with using AI in my work and/or daily life.* | 1100 (40.6%) | 423 (31.1%) | 677 (50.1%) |  |
| Disagree  *I rarely use AI in my work and/or daily life.* | 179 (6.6%) | 72 (5.3%) | 107 (7.9%) |  |
| Strongly disagree  *I rarely use AI in my work and/or daily life AND I would rather I have nothing to do with AI.* | 21 (0.8%) | 5 (0.4%) | 16 (1.2%) |  |
| **Q10. I am experienced with using medical AI in my clinical practice** |  |  |  | <0.0001 ^b^ |
| Strongly agree  *NOT ONLY am I good at using medical AI BUT ALSO I constantly and actively learn new skills to expand my toolbox for medical AI.* | 308 (11.4%) | 239 (17.6%) | 69 (5.1%) |  |
| Agree  *I use medical AI AND I am good at using medical AI tool(s).* | 741 (27.4%) | 460 (33.9%) | 281 (20.8%) |  |
| Neutral  *I have some experience with using medical AI.* | 1262 (46.6%) | 521 (38.4%) | 741 (54.9%) |  |
| Disagree  *I rarely use medical AI.* | 357 (13.2%) | 130 (9.6%) | 227 (16.8%) |  |
| Strongly disagree  *I rarely use medical AI AND I would rather I have nothing to do with medical AI.* | 40 (1.5%) | 8 (0.6%) | 32 (2.4%) |  |
| **Q11. I believe AI eventually will transform the healthcare industry** |  |  |  | <0.0001 ^b^ |
| Strongly agree  *I believe AI eventually will dramatically transform healthcare AND moreover any physician who refuses to “ride the wave” would become outdated.* | 666 (24.6%) | 454 (33.4%) | 212 (15.7%) |  |
| Agree  *I believe AI eventually will dramatically transform healthcare, but some “conventional” physicians who do not use AI can still find their niche.* | 1285 (47.5%) | 628 (46.2%) | 657 (48.7%) |  |
| Neutral  *I believe AI eventually will transform healthcare to some extent, and not all physicians need to learn to use medical AI.* | 695 (25.7%) | 260 (19.1%) | 435 (32.2%) |  |
| Disagree  *I believe there will be limited niche applications of AI in medicine, AND healthcare will continue to rely on “conventional” physicians who do not utilize or depend on medical AI.* | 52 (1.9%) | 14 (1.0%) | 38 (2.8%) |  |
| Strongly disagree  *I believe healthcare will continue to rely on “conventional” physicians who do not utilize or depend on medical AI. I believe medical AI is hyped and, like other hypes before it, it will fail to deliver its promise.* | 10 (0.4%) | 2 (0.1%) | 8 (0.6%) |  |
| **Q12. I believe AI eventually will augment physicians’ capabilities** |  |  |  | <0.0001 ^b^ |
| Strongly agree  *I believe judicious use of medical AI will make most physicians better physicians and better able to care for patients, AND moreover most physicians will better love their jobs because of medical AI.* | 702 (25.9%) | 457 (33.7%) | 245 (18.1%) |  |
| Agree  *I believe judicious use of medical AI will make most physicians better physicians and better able to care for patients.* | 1232 (45.5%) | 627 (46.2%) | 605 (44.8%) |  |
| Neutral  *I believe medical AI will augment capabilities of some but not all physicians.* | 712 (26.3%) | 259 (19.1%) | 453 (33.6%) |  |
| Disagree  *For most physicians I believe medical AI will not augment their capability to care for patients.* | 44 (1.6%) | 13 (1.0%) | 31 (2.3%) |  |
| Strongly disagree  *For most physicians I believe medical AI will not augment their capability to care for patients AND moreover, if medical AI becomes common in clinical practice, I believe* *many physicians will not enjoy their jobs as much as they do today.* | 18 (0.7%) | 2 (0.1%) | 16 (1.2%) |  |
| **Q13. I believe AI eventually will improve healthcare quality** |  |  |  | <0.0001 ^b^ |
| Strongly agree  *With judicious use of medical AI, I believe most patients will be better cared for and attain better health AND moreover most patients will be more satisfied with the healthcare they receive.* | 694 (25.6%) | 449 (33.1%) | 245 (18.1%) |  |
| Agree  *With judicious use of medical AI, I believe most patients will be better cared for and attain better health.* | 1266 (46.8%) | 649 (47.8%) | 617 (45.7%) |  |
| Neutral  *I believe medical AI will improve the quality or expediency of healthcare for some patients but lower the quality or expediency for others.* | 698 (25.8%) | 249 (18.3%) | 449 (33.3%) |  |
| Disagree  *For most patients, I believe medical AI would not make meaningful and positive impact on their healthcare.* | 39 (1.4%) | 8 (0.6%) | 31 (2.3%) |  |
| Strongly disagree  *For most patients, I believe medical AI would not make meaningful and positive impact on their healthcare AND moreover, if medical AI becomes common in clinical practice, I believe many patients will become less satisfied with the healthcare they receive.* | 11 (0.4%) | 3 (0.2%) | 8 (0.6%) |  |
| **Q14. I believe AI eventually will improve equity in access to healthcare** |  |  |  | <0.0001 ^b^ |
| Strongly agree  *NOT ONLY I believe medical AI will make healthcare more equitable and promote population health, BUT ALSO I believe medical AI is one of the most important tools for achieving common prosperity.* | 620 (22.9%) | 417 (30.7%) | 203 (15.0%) |  |
| Agree  *I believe medical AI will make healthcare more equitable and promote population health.* | 1192 (44%) | 629 (46.3%) | 563 (41.7%) |  |
| Neutral  *I believe medical AI is only one of the many tools we could try to test whether they could promote population health.* | 859 (31.7%) | 306 (22.5%) | 553 (41.0%) |  |
| Disagree  *I believe medical AI will not make healthcare more equitable AND it will not promote population health.* | 25 (0.9%) | 5 (0.4%) | 20 (1.5%) |  |
| Strongly disagree  *NOT ONLY I believe medical AI will not make healthcare more equitable, BUT ALSO I believe medical AI initiatives will divert crucial resources from other more impactful tools for promoting population health.* | 12 (0.4%) | 1 (0.1%) | 11 (0.8%) |  |
| **Q15. I believe medical AI eventually will facilitate physicians’ training** |  |  |  | <0.0001 ^b^ |
| Strongly agree  *NOT ONLY I believe medical AI eventually will expedite the training of new physicians, BUT ALSO I believe, because of medical AI, new waves of physicians will be better trained compared with trainees in conventional training programs.* | 656 (24.2%) | 434 (32.0%) | 222 (16.4%) |  |
| Agree  *I believe medical AI will expedite the training of new physicians.* | 1191 (44%) | 636 (46.8%) | 555 (41.1%) |  |
| Neutral  *I believe medical AI will facilitate some but not all aspects of physician training.* | 781 (28.8%) | 277 (20.4%) | 504 (37.3%) |  |
| Disagree  *I believe reliance on medical AI will interfere with junior physicians’ learning journey and slow down their learning.* | 64 (2.4%) | 9 (0.7%) | 55 (4.1%) |  |
| Strongly disagree  *I believe reliance on medical AI will interfere with junior physicians’ learning journey, slow down their learning, AND lead to new waves of under-qualified physicians.* | 16 (0.6%) | 2 (0.1%) | 14 (1.0%) |  |
| **Q16. When considering medical AI, which of the following is more important to me? (Choose one.)** |  |  |  | <0.0001 ^a^ |
| A medical AI system is efficacious in delivering what it promises | 926 (34.2%) | 540 (39.8%) | 386 (28.6%) |  |
| A medical AI system does not affect my autonomy as a physician | 1782 (65.8%) | 818 (60.2%) | 964 (71.4%) |  |
| **Q17. I think AI prescription might be useful in the following situations: (Choose ≤3 options that best describe what I think.)** |  |  |  |  |
| When clinical guidelines clearly specify standardized treatment plans | 1995 (73.7%) | 1097 (80.8%) | 898 (66.5%) | <0.0001 ^a^ |
| When refilling drug prescription that is already known to be safe in a given patient | 1496 (55.2%) | 778 (57.3%) | 718 (53.2%) | 0.03 ^a^ |
| When there are too many clinical factors to consider before making a decision on drug prescription | 1198 (44.2%) | 611 (45.0%) | 587 (43.5%) | 0.45 ^a^ |
| When it is an unusual occasion wherein total reliance on humans may cause delay in starting therapy or lapse in decision-making | 660 (24.4%) | 281 (20.7%) | 379 (28.1%) | <0.0001 ^a^ |
| When the AI system explains the rationale underlying the prescription it recommends | 461 (17%) | 171 (12.6%) | 290 (21.5%) | <0.0001 ^a^ |
| When physicians need to focus on other, more important clinical decisions | 426 (15.7%) | 168 (12.4%) | 258 (19.1%) | <0.0001 ^a^ |
| When there is a shortage of qualified staffs | 291 (10.7%) | 146 (10.8%) | 145 (10.7%) | >0.99 ^a^ |
| None of the above | 46 (1.7%) | 16 (1.2%) | 30 (2.2%) | 0.051 ^a^ |
| **Q18. Which of the following aspects of efficacy-vetting are the most important for accepting the use of an AI prescription model? (Choose ≤2 options that best describe what I think.)** |  |  |  |  |
| The AI model is developed using data from cohorts that are similar to my patients. | 1306 (48.2%) | 860 (63.3%) | 446 (33.0%) | <0.0001 ^a^ |
| The model has been validated in ≥1 domestic cohort. | 1068 (39.4%) | 415 (30.6%) | 653 (48.4%) | <0.0001 ^a^ |
| The model has been validated in ≥1 independent cohort. | 734 (27.1%) | 479 (35.3%) | 255 (18.9%) | <0.0001 ^a^ |
| The model’s test result has been published in a highly-regarded peer-reviewed journal. | 722 (26.7%) | 272 (20.0%) | 450 (33.3%) | <0.0001 ^a^ |
| The model is endorsed by ≥1 reputable international medical professional society. | 540 (19.9%) | 205 (15.1%) | 335 (24.8%) | <0.0001 ^a^ |
| The model is endorsed by ≥1 reputable domestic medical professional society. | 423 (15.6%) | 152 (11.2%) | 271 (20.1%) | <0.0001 ^a^ |
| **Q19. How would I define “expediency” of an AI prescription model? (Choose ≤3 options that best describe what I think.)** |  |  |  |  |
| The model can be embedded in the workflow of my clinical practice. | 1645 (60.7%) | 1013 (74.6%) | 632 (46.8%) | <0.0001 ^a^ |
| The model would enable me to shorten my work hours while maintaining quality of care. | 1324 (48.9%) | 529 (39.0%) | 795 (58.9%) | <0.0001 ^a^ |
| The model would enable me to grow my clinical practice while maintaining quality of care. | 1099 (40.6%) | 579 (42.6%) | 520 (38.5%) | 0.03 ^a^ |
| The model would not increase my work load. | 1056 (39%) | 625 (46.0%) | 431 (31.9%) | <0.0001 ^a^ |
| The model could help me mitigate the risk of making errors. | 624 (23%) | 209 (15.4%) | 415 (30.7%) | <0.0001 ^a^ |
| The model could augment my capability for decision-making. | 405 (15%) | 123 (9.1%) | 282 (20.9%) | <0.0001 ^a^ |
| The model would allow me to focus on other activities that matter. | 394 (14.5%) | 151 (11.1%) | 243 (18.0%) | <0.0001 ^a^ |
| The model would make it easier for me to hire and train junior physicians. | 102 (3.8%) | 27 (2.0%) | 75 (5.6%) | <0.0001 ^a^ |
| The model would improve patients’ trust in me. | 29 (1.1%) | 12 (0.9%) | 17 (1.3%) | 0.45 ^a^ |
| **Q20. Before I decide to use an AI prescription model in my clinical practice, technical details regarding how the model was built and validated (even if not everyone can understand them) need to be transparent.** |  |  |  | <0.0001 ^b^ |
| Strongly agree  *Before I can decide whether to use an AI prescription model in my clinical practice, I personally need to know all the technical details related to model construction including training data, model-fitting, performance metrics, and validation data.* | 1079 (39.8%) | 610 (44.9%) | 469 (34.7%) |  |
| Agree  *I only need to know a portion of technical details related to model construction before I decide whether to use the model in my clinical practice.* | 1105 (40.8%) | 561 (41.3%) | 544 (40.3%) |  |
| Neutral  *Whether the technical details of AI model construction are accessible to me is irrelevant to my decision on whether to adopt an AI prescription model in my clinical practice.* | 495 (18.3%) | 179 (13.2%) | 316 (23.4%) |  |
| Disagree  *Whether the technical details of AI model construction are accessible to me is irrelevant to my decision on whether to adopt an AI prescription model in my clinical practice. All I need to know is that the AI prescription model has been vetted by experts, and there is NO need for the model’s technical details to be made fully transparent after review by experts.* | 22 (0.8%) | 6 (0.4%) | 16 (1.2%) |  |
| Strongly disagree  *Whether the technical details of AI model construction are accessible to me is irrelevant to my decision on whether to adopt an AI prescription model in my clinical practice. All I need to know is that the AI prescription model has been vetted by experts, and there is NO need for the model’s technical details to be made fully transparent after review by experts. MOREOVER, I believe mandatory full disclosure of proprietary information might discourage innovation and harm societal progress.* | 7 (0.3%) | 2 (0.1%) | 5 (0.4%) |  |
| **Q21. Which of the following aspects of an AI prescription model’s “explainability” are the most important to me? (Choose ≤2 options that best describe what I think.)** |  |  |  |  |
| I understand how the AI model converts input variables to prescriptions. | 1275 (47.1%) | 775 (57.1%) | 500 (37.0%) | <0.0001 ^a^ |
| When the AI model’s recommendation is discordant with my opinion, the model can explain to me its reasoning. | 1273 (47%) | 397 (29.2%) | 876 (64.9%) | <0.0001 ^a^ |
| I know and agree with the model’s input variables. | 700 (25.8%) | 526 (38.7%) | 174 (12.9%) | <0.0001 ^a^ |
| I am able to explain to my patients how the model works. | 679 (25.1%) | 223 (16.4%) | 456 (33.8%) | <0.0001 ^a^ |
| I am able to explain to my colleagues how the model works. | 475 (17.5%) | 256 (18.9%) | 219 (16.2%) | 0.08 ^a^ |
| **Q22. Which of the following aspects of governance/stewardship of AI prescription are the most important to me? (Choose ≤2 options that best describe what I think.)** |  |  |  |  |
| There is a trustworthy mechanism for monitoring quality, bias, and safety of AI prescriptions. | 1468 (54.2%) | 544 (40.1%) | 924 (68.4%) | <0.0001 ^a^ |
| There is a trustworthy mechanism for the model’s maintenance and/or upgrades. | 1244 (45.9%) | 817 (60.2%) | 427 (31.6%) | <0.0001 ^a^ |
| I know whom to contact when I encounter problem(s) and/or need help with the AI prescription model. | 1167 (43.1%) | 701 (51.6%) | 466 (34.5%) | <0.0001 ^a^ |
| There is an auditing mechanism for upholding the AI model’s adherence to data privacy and security. | 714 (26.4%) | 196 (14.4%) | 518 (38.4%) | <0.0001 ^a^ |
| **Q23. Which of the following technological attributes of an AI prescription model are the most important to me? (Rank the following items from the most important [rank = 1] to the least important [rank = 5].)** |  |  |  |  |
| Expediency | 1.9 ^§^ | 1.9 ^§^ | 2.0 ^§^ | 0.89 ^b^ |
| Vetted efficacy | 2.0 ^§^ | 1.8 ^§^ | 2.2 ^§^ | <0.0001 ^b^ |
| Explainability | 3.5 ^§^ | 3.6 ^§^ | 3.4 ^§^ | <0.0001 ^b^ |
| Governance/stewardship | 3.7 ^§^ | 4.0 ^§^ | 3.5 ^§^ | <0.0001 ^b^ |
| Transparency | 3.8 ^§^ | 3.7 ^§^ | 3.9 ^§^ | <0.0001 ^b^ |
| **Q24. In my opinion, which of the following aspects of institutional culture would be the most important for successful adoption of AI prescription at my institution? (Choose ≤4 options that best describe what I think.)** |  |  |  |  |
| My hospital embraces new technologies. | 1712 (63.2%) | 1032 (76.0%) | 680 (50.4%) | <0.0001 ^a^ |
| My hospital values innovation. | 1711 (63.2%) | 1090 (80.3%) | 621 (46.0%) | <0.0001 ^a^ |
| My hospital is willing to invest resources in AI initiatives. | 1370 (50.6%) | 790 (58.2%) | 580 (43.0%) | <0.0001 ^a^ |
| My hospital values employee satisfaction and interpersonal harmony. | 1358 (50.1%) | 757 (55.7%) | 601 (44.5%) | <0.0001 ^a^ |
| My hospital promotes learning and talent development. | 868 (32.1%) | 258 (19.0%) | 610 (45.2%) | <0.0001 ^a^ |
| My hospital is team-oriented and values collaboration. | 747 (27.6%) | 302 (22.2%) | 445 (33.0%) | <0.0001 ^a^ |
| My hospital values stability and continuity. | 376 (13.9%) | 110 (8.1%) | 266 (19.7%) | <0.0001 ^a^ |
| My hospital upholds transparent communications to employees. | 363 (13.4%) | 119 (8.8%) | 244 (18.1%) | <0.0001 ^a^ |
| My hospital focuses on achieving measurable goals and celebrates success. | 223 (8.2%) | 62 (4.6%) | 161 (11.9%) | <0.0001 ^a^ |
| My hospital is receptive to changes of workflows. | 204 (7.5%) | 72 (5.3%) | 132 (9.8%) | <0.0001 ^a^ |
| **Q25. In my opinion, adoption of AI prescription at my institution would be more likely to be successful if _____ (Choose ≤3 options that best describe what I think.)** |  |  |  |  |
| There is a dedicated team of local “champions” to ensure successful implementation of AI prescription models. | 1576 (58.2%) | 875 (64.4%) | 701 (51.9%) | <0.0001 ^a^ |
| The leadership team at my hospital supports the adoption of AI in prescription. | 1498 (55.3%) | 981 (72.2%) | 517 (38.3%) | <0.0001 ^a^ |
| My hospital provides guidance on the use of AI in prescription. | 1482 (54.7%) | 909 (66.9%) | 573 (42.4%) | <0.0001 ^a^ |
| Planning of the AI prescription initiative involves a multi-disciplinary team. | 887 (32.8%) | 266 (19.6%) | 621 (46.0%) | <0.0001 ^a^ |
| Planning of the AI prescription initiative is transparent. | 710 (26.2%) | 308 (22.7%) | 402 (29.8%) | <0.0001 ^a^ |
| There is a plan for educating and training the staffs on the AI prescription model’s use. | 522 (19.3%) | 119 (8.8%) | 403 (29.9%) | <0.0001 ^a^ |
| Planning of the AI prescription initiative consults or involves me. | 151 (5.6%) | 51 (3.8%) | 100 (7.4%) | <0.0001 ^a^ |
| **Q26. In my opinion, which of the following aspects of information technology (IT) and data science resource availability and/or access are the most important for successful adoption of AI prescription at my institution? (Rank the following items from the most important [rank = 1] to the least important [rank = 3].)** |  |  |  |  |
| A reliable and capable in-house IT/data science specialist team. | 1.7 ^§^ | 1.5 ^§^ | 2.0 ^§^ | <0.0001 ^b^ |
| A reliable and capable 3^rd^-party partner(s) or vendor(s). | 2.1 ^§^ | 2.0 ^§^ | 2.2 ^§^ | <0.0001 ^b^ |
| Employees from disciplines other than IT/data science are also held responsible for successful introduction of the new technology. | 2.2 ^§^ | 2.5 ^§^ | 1.9 ^§^ | <0.0001 ^b^ |
| **Q27. In my opinion, after successful adoption of AI prescription, my institution will need to improve _____ to maintain or further its edge compared to other medical institutions. (Choose ≤3 options that best describe what I think.)** |  |  |  |  |
| Ability of specialists from multiple disciplines to work collaboratively to deliver patient care. | 1719 (63.5%) | 686 (50.5%) | 1033 (76.5%) | <0.0001 ^a^ |
| Cost-effectiveness. | 1278 (47.2%) | 856 (63.0%) | 422 (31.3%) | <0.0001 ^a^ |
| Throughput. | 1246 (46%) | 909 (66.9%) | 337 (25.0%) | <0.0001 ^a^ |
| Expertise to manage diseases that are difficult to treat. | 1093 (40.4%) | 363 (26.7%) | 730 (54.1%) | <0.0001 ^a^ |
| Integration of clinical practice with research to accelerate the advent of next-generation treatments. | 834 (30.8%) | 233 (17.2%) | 601 (44.5%) | <0.0001 ^a^ |
| Empathy and caregiving. | 832 (30.7%) | 600 (44.2%) | 232 (17.2%) | <0.0001 ^a^ |
| Capability to care for patients who have special needs. | 583 (21.5%) | 201 (14.8%) | 382 (28.3%) | <0.0001 ^a^ |
| **Q28. In my opinion, which of the following is the most important for successful adoption of AI prescription in my clinical practice? (Rank the following items from the most important [rank = 1] to the least important [rank = 3].)** |  |  |  |  |
| There is a nation-wide initiative to promote the use of AI in healthcare. | 1.9 ^§^ | 1.5 ^§^ | 2.2 ^§^ | <0.0001 ^b^ |
| There is a nation-wide initiative to improve technological infrastructure for medical AI. | 1.9 ^§^ | 1.9 ^§^ | 1.9 ^§^ | 0.53 ^b^ |
| There is a nation-wide initiative to educate/train talents for medical AI. | 2.2 ^§^ | 2.5 ^§^ | 1.9 ^§^ | <0.0001 ^b^ |
| **Q29. In my opinion, who should set the standards for AI prescription systems? Choose one that best applies.** |  |  |  | <0.0001 ^a^ |
| Regulatory authorities. | 169 (6.2%) | 123 (9.1%) | 46 (3.4%) |  |
| Professional medical societies. | 328 (12.1%) | 229 (16.9%) | 99 (7.3%) |  |
| Each healthcare organization should set its own standard. | 272 (10%) | 188 (13.8%) | 84 (6.2%) |  |
| Regulatory authorities + Professional medical societies | 371 (13.7%) | 195 (14.4%) | 176 (13.0%) |  |
| Regulatory authorities + Each healthcare organization | 230 (8.5%) | 153 (11.3%) | 77 (5.7%) |  |
| Professional medical societies + Each healthcare organization | 247 (9.1%) | 111 (8.2%) | 136 (10.1%) |  |
| Regulatory authorities + Professional medical societies + Each healthcare organization | 1091 (40.3%) | 359 (26.4%) | 732 (54.2%) |  |
| **Q30. In my opinion, who should be remunerated when an AI model is utilized for prescribing a drug (that is, additional charge beyond drug price)? Choose one that best applies.** |  |  |  | <0.0001 ^a^ |
| No one. | 445 (16.4%) | 186 (13.7%) | 259 (19.2%) |  |
| The hospital that has invested in the new AI technology. | 516 (19.1%) | 320 (23.6%) | 196 (14.5%) |  |
| The physician who uses the model and reviews its output. | 356 (13.1%) | 227 (16.7%) | 129 (9.6%) |  |
| The developer who built and maintains/updates the model. | 361 (13.3%) | 181 (13.3%) | 180 (13.3%) |  |
| Hospital + Physician | 170 (6.3%) | 88 (6.5%) | 82 (6.1%) |  |
| Hospital + Developer | 251 (9.3%) | 101 (7.4%) | 150 (11.1%) |  |
| Physician + Developer | 74 (2.7%) | 39 (2.9%) | 35 (2.6%) |  |
| Hospital + Physician + Developer | 535 (19.8%) | 216 (15.9%) | 319 (23.6%) |  |
| **Q31. I anticipate ≥1 physician (possibly including myself) at my hospital will become ready to utilize AI to prescribe a drug within _____ years.** |  |  |  | <0.0001 ^b^ |
| ≤1 | 398 (14.7%) | 244 (18.0%) | 154 (11.4%) |  |
| 1.1 – 3 | 1042 (38.5%) | 555 (40.9%) | 487 (36.1%) |  |
| 3.1 – 5 | 784 (29%) | 361 (26.6%) | 423 (31.3%) |  |
| 5.1 – 10 | 347 (12.8%) | 162 (11.9%) | 185 (13.7%) |  |
| >10 | 108 (4%) | 30 (2.2%) | 78 (5.8%) |  |
| Never | 29 (1.1%) | 6 (0.4%) | 23 (1.7%) |  |
| **Q32. I anticipate I myself will become ready to utilize AI to prescribe a drug within _____ years.** |  |  |  | <0.0001 ^b^ |
| ≤1 | 358 (13.2%) | 221 (16.3%) | 137 (10.1%) |  |
| 1.1 – 3 | 952 (35.2%) | 514 (37.8%) | 438 (32.4%) |  |
| 3.1 – 5 | 807 (29.8%) | 372 (27.4%) | 435 (32.2%) |  |
| 5.1 – 10 | 413 (15.3%) | 199 (14.7%) | 214 (15.9%) |  |
| >10 | 135 (5%) | 40 (2.9%) | 95 (7.0%) |  |
| Never | 43 (1.6%) | 12 (0.9%) | 31 (2.3%) |  |

Items in Q17, Q18, Q19, Q21, Q22, Q23, Q24, Q25, Q26, Q27, and Q28 are ordered according to their frequencies of being selected by the respondents.

^§^ Average ranking (highest rank = 1).

^†^ Median (inter-quartile range).

^‡^ A tier-1 hospital in the Chinese mainland is equivalent to a community clinical service center in the US whilst a tier-2 hospital is a mid-sized hospital and a tier-3 hospital, a large-sized hospital or an academic medical center.

^a^ Chi-squared test.

^b^ Two-tailed Wilcoxon test.

**Supplement Figure 1. Results of 1000 bootstrapping experiments.**
